# *Plasmodium falciparum* HSP90 inhibitors show divergent resistance despite a shared ATP-binding site

**DOI:** 10.1016/j.celrep.2026.117664

**Published:** 2026-07-05

**Authors:** Fu-Hsuan Ko, Amanda K. Lukens, Mariana Laureano de Souza, Alex Foy, Jason Hsiao, Tolla Ndiaye, John Okombo, Tomas Yeo, Heekuk Park, Anne-Catrin Uhlemann, Nonlawat Boonyalai, Krittikorn Kumpornsin, Gareth Girling, Charisse Pasaje, Luiz Godoy, Priyan Kapoor, Gregory L. Durst, Sabine Ottilie, Arnab Chatterjee, Jacquin Niles, Marcus Lee, David A. Fidock, Dyann F. Wirth, Elizabeth A. Winzeler

**Affiliations:** 1 Division of Biological Sciences, University of California, La Jolla, San Diego, CA, USA; 2 Department of Pediatrics, University of California, San Diego, San Diego School of Medicine, La Jolla, San Diego, CA, USA; 3 Infectious Disease and Microbiome Program, The Broad Institute, Cambridge, MA, USA; 4 Department of Immunology and Infectious Diseases, Harvard T.H. Chan School of Public Health, Boston, MA, USA; 5 Department of Microbiology and Immunology, Columbia University Irving Medical Center, New York, NY, USA; 6 Center for Malaria Therapeutics and Antimicrobial Resistance, Division of Infectious Diseases, Department of Medicine, Columbia University Irving Medical Center, New York, NY 10032, USA; 7 Biological Chemistry and Drug Discovery, Wellcome Centre for Anti-Infectives Research, University of Dundee, Dundee, UK; 8 Wellcome Sanger Institute, Wellcome Genome Campus, Hinxton, Cambridgeshire, UK; 9 Department of Biological Engineering, Massachusetts Institute of Technology, Cambridge, MA, USA; 10 Department of Biological Engineering, University of California, La Jolla, San Diego, CA, USA; 11 Lgenia Inc., Fortville, IN 46040, USA; 12 Scripps Research Institute, La Jolla, CA 92037, USA; 13 Lead contact

## Abstract

Drug resistance poses a major challenge across therapeutic areas including malaria, yet factors governing resistance propensity remain poorly understood. We demonstrate that two HSP90 inhibitors targeting the identical ATP-binding site exhibit dramatically different resistance profiles in *P. falciparum*. Geldanamycin readily selected 10 distinct resistance mutations conferring up to 22-fold resistance, while AUY-922 required 44 weeks to yield a single A41S mutation with only 2-fold resistance. Resistance mutations mapped throughout geldanamycin’s binding pocket but localized near the ATP-binding site for AUY-922. Strikingly, A41S enhanced AUY-922 binding affinity via additional hydrogen bonding, yet stronger binding paradoxically correlated with resistance. Conditional HSP90 knockdown increased geldanamycin sensitivity but not AUY-922 activity, indicating distinct target dependencies despite shared binding sites. We propose AUY-922’s lower resistance risk reflects engagement of multiple HSP90 family members. These findings demonstrate that resistance risk cannot be predicted from binding site identity alone, informing development of more durable therapeutics.

## In brief

Ko et al. demonstrate that two *Plasmodium falciparum* heat shock protein 90 inhibitors targeting the identical binding site exhibit strikingly divergent resistance profiles. A paradoxical resistance mutation that strengthens rather than weakens drug binding reveals that resistance risk cannot be inferred from binding site identity alone.

## Graphical Abstract

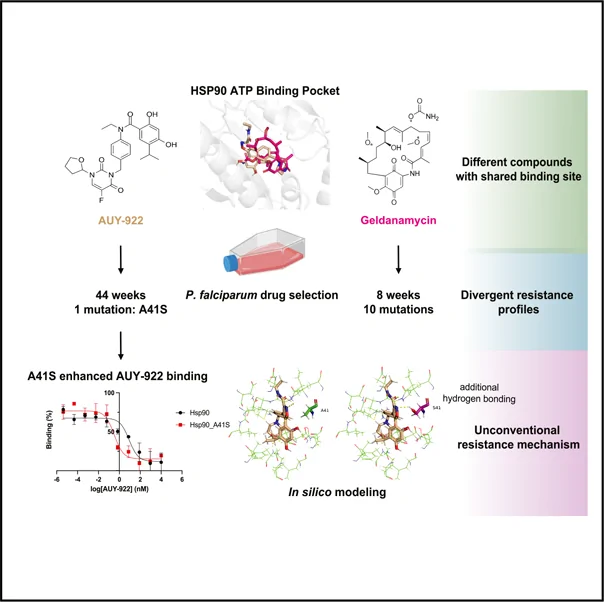

## INTRODUCTION

Drug resistance threatens therapeutic efficacy across multiple medical fields, with malaria representing a critical example where resistance evolution undermines treatment success. *Plasmodium falciparum* (*P. falciparum*) causes the most severe malaria cases, and despite extensive control efforts, the disease continues to affect millions annually with hundreds of thousands of deaths.^1^ Resistance has emerged against all deployed antimalarial drugs within years to decades of introduction, creating cycles of drug development and resistance that threaten elimination goals.^2,3^ This creates an urgent need for new therapeutic approaches that can overcome existing resistance mechanisms and ideally exhibit enhanced durability against future resistance evolution.^4^ Current antimalarial drug discovery efforts emphasize the need to address fundamental questions in drug development: can resistance risk be predicted from target characteristics and do certain design approaches yield compounds with inherently higher barriers to resistance evolution? Understanding these principles has implications beyond malaria for any therapeutic area where drug resistance threatens treatment durability.

Heat shock protein 90 (HSP90) has attracted attention as a potential antimalarial target due to its essential role as a molecular chaperone. As a highly conserved ATPase, HSP90 is involved in the folding, maturation, and maintenance of numerous client proteins critical for cellular function, making it an attractive target for therapeutic intervention.^5,6^ HSP90 protein plays crucial roles in protein quality control, signal transduction, and stress response pathways that are essential for parasite survival and development. *Plasmodium* parasites express multiple essential HSP90 isoforms in different organelles, including the (endoplasmic reticulum) ER-resident GRP94 (glucose-regulated protein 94), which may offer additional therapeutic opportunities for targeting. Several HSP90 inhibitors have demonstrated potent antimalarial activity against *P. falciparum* with half maximal inhibitory concentration (IC_50_) values often in the nanomolar range.^7,8^ Some of these compounds were identified from the Calibr REFRAME collection, a library of small molecules that have reached clinical development or undergone significant preclinical profiling.^9^ These include AUY922 (luminespib), which had previously advanced to human clinical trials for cancer treatment. However, the precise mechanisms by which these inhibitors achieve their antimalarial effects and their susceptibility to resistance development remain incompletely characterized.

Most HSP90 inhibitors target the highly conserved ATP-binding pocket, leading to the expectation that compounds binding to this site would exhibit similar resistance profiles. The ATP-binding domain is structurally conserved across species^10^ and represents the most commonly targeted site for HSP90 inhibition.^11^ While this conservation might suggest similar resistance mechanisms across species, resistance development can be more complex. Resistance can arise through multiple mechanisms beyond target binding, including altered drug kinetics, cellular distribution, and protein function. Understanding how these factors influence resistance development is crucial for predicting the useful therapeutic lifespan of HSP90-targeted antimalarials and for designing compounds with improved resistance profiles.

We employed comprehensive resistance profiling including *in vitro* resistance evolution experiments, minimum inoculum for resistance assays, CRISPR-Cas9 gene editing, conditional knockdown studies, and biophysical binding analysis. Surprisingly, we found that despite targeting the same binding site, these compounds exhibited dramatically different resistance profiles, with AUY-922 showing an exceptionally high barrier to resistance development compared to geldanamycin (GA). We identify a paradoxical resistance mechanism where enhanced drug binding correlates with reduced antimalarial efficacy, challenging conventional structure-activity relationships. These findings demonstrate that resistance risk cannot be predicted solely from target binding site identity and reveal how compounds with similar binding modes can exhibit different evolutionary vulnerabilities, with implications for designing more durable antimalarial therapies.

## RESULTS

### Evaluation of two structurally distinct HSP90 inhibitors

**Figure 1.**
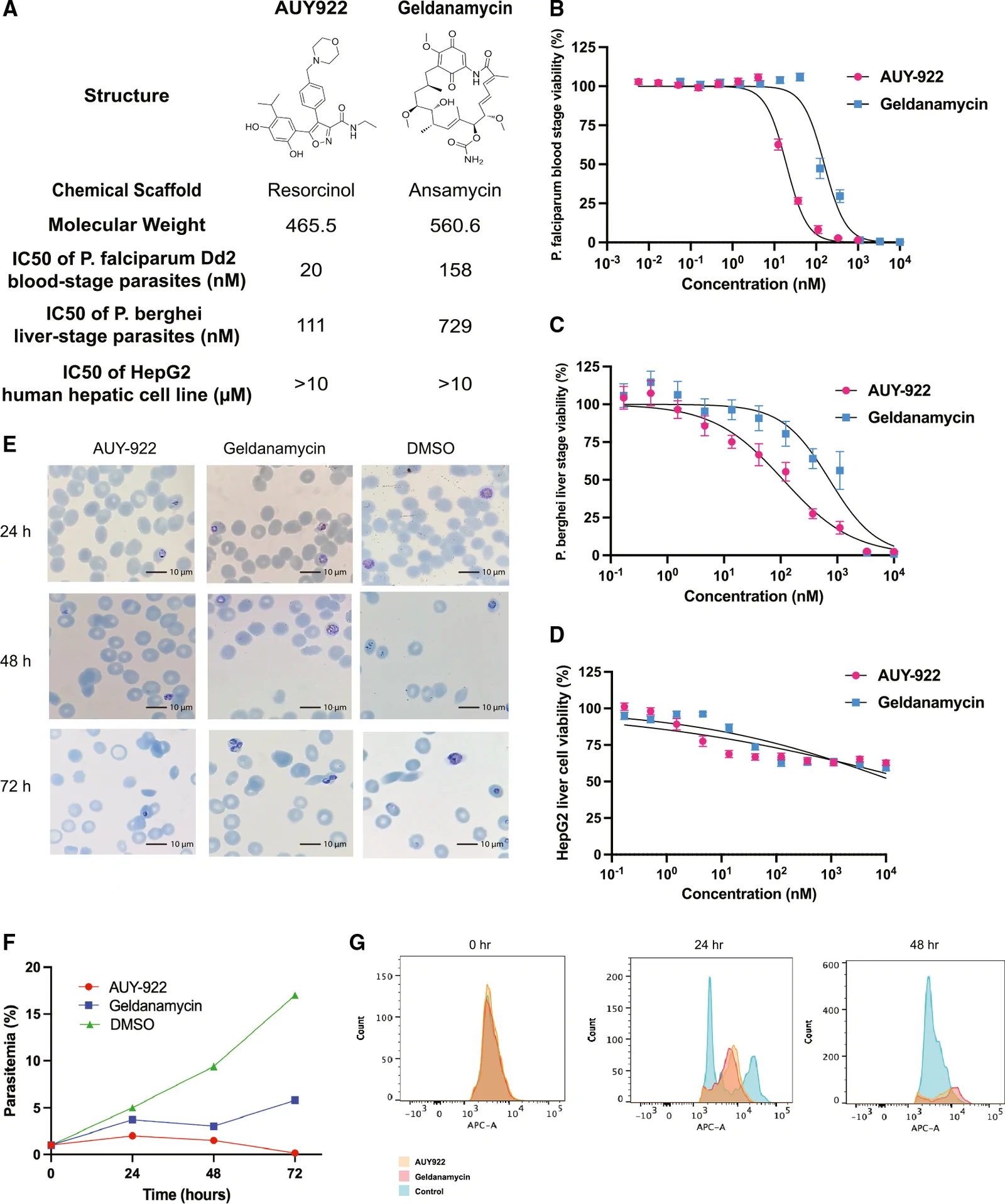
Characterization and mechanism of action studies of HSP90 inhibitors (A) Chemical structures and biological activity profiles of two structurally diverse HSP90 inhibitors: AUY-922 (resorcinol-based) and geldanamycin (ansamycin), summarizing blood stage, liver stage, and cytotoxicity data from (B–D). (B) Dose-response curves for AUY-922 and geldanamycin against *P. falciparum* Dd2 strain. IC_50_ values were determined using a 72-h SYBR green-based growth assay. (C) Life stage-specific activity assessment showing IC_50_ values of AUY-922 and geldanamycin against liver stage *P. berghei* parasites in a luciferase-based assay. (D) Cytotoxicity assessment in HepG2 human hepatocytes showing that both AUY-922 and geldanamycin exhibited minimal toxicity, with no measurable IC_50_ values obtained at concentrations up to 10 μM. (E) Giemsa-stained blood smears showing parasite morphology following drug treatment. Synchronized ring-stage *P. falciparum* at 1% parasitemia were treated with DMSO control, AUY-922, or geldanamycin at 5× IC_50_. Images were taken at 24, 48, and 72 h post-treatment. Scale bars, 10 μm. (F) Parasitemia growth curves from the same experiment shown in (E). Parasitemia was measured every 24h over 72 h for AUY-922, geldanamycin, and DMSO control. (G) Flow cytometry analysis using SYTO61 DNA staining showing intraerythrocytic developmental stages of *P. falciparum* after drug treatment. Histograms demonstrate parasite progression through the blood stage life cycle, with distinct profiles for ring, trophozoite, and schizont stages. Data are represented as mean ± SEM of biological replicates. See also Figure S1.

We first evaluated two structurally distinct HSP90 inhibitors that bind to the ATP-binding pocket against asexual blood-stage *P. falciparum* Dd2 parasites (Figure 1A). AUY-922 (luminespib) is a resorcinol-based inhibitor that was originally developed for cancer treatment and reached phase 2 clinical trials,^12^ while GA is an ansamycin antibiotic natural product that has served as a prototype HSP90 inhibitor. Despite targeting the same binding site, AUY-922 showed superior potency (IC_50_ = 20 nM) compared to GA (IC_50_ = 158 nM), an ~8-fold difference in activity (Figure 1B). To assess their potential as multi-stage antimalarials, we evaluated activity against liver-stage parasites using a *Plasmodium berghei* (*P. berghei*) luciferase reporter system.^13^ AUY-922 maintained its potency advantage in liver stages (IC_50_ = 111 nM) compared to GA (IC_50_ = 729 nM) (Figure 1C). Neither compound exhibited significant cytotoxicity against human HepG2 hepatocytes at concentrations up to 10 μM, providing initial selectivity data for these HSP90 inhibitors in the hepatocyte model system (Figure 1D). We also characterized radicicol, a resorcylic acid lactone natural product that targets the same ATP-binding pocket as AUY-922 and GA. Radicicol demonstrated antimalarial activity against blood-stage *P. falciparum* (IC_50_ = 1.91 μM) and liver-stage *P. berghei* parasites (IC_50_ = 13.92 μM), with cytotoxicity against human hepatocytes at higher concentrations (IC_50_ = 8.05 μM) (Figure S1). This third HSP90 inhibitor provided additional validation of the target and served as a useful comparator in subsequent resistance and conditional knockdown experiments.

To assess the antiparasitic activity of AUY-922 and GA, tightly synchronized ring-stage parasites at 1% parasitemia were treated with 5× IC_50_ concentrations of either compound and monitored over 72 h. Parasitemia was measured every 24 h by Giemsa-stained blood smear (Figure 1E). At 24 h, dimethyl sulfoxide (DMSO) control parasites had progressed to schizont stage as expected, while both AUY-922- and GA-treated parasites failed to complete this developmental transition. At 48 h, AUY-922-treated parasites displayed abnormal morphology including pyknotic forms and parasites outside red blood cells, while GA-treated cultures contained a small proportion of morphologically normal parasites, suggesting partial survival under drug pressure. By 72 h, AUY-922-treated cultures showed complete parasite clearance, while GA-treated parasites exhibited a modest increase in parasitemia compared to 48 h, indicating some degree of parasite recovery (Figure 1F). Flow cytometry with SYTO61 DNA staining further supported these observations (Figure 1G).^14^ At 24 h, DMSO control parasites exhibited a characteristic dual-peak pattern reflecting normal schizont development and nuclear division, while both AUY-922- and GA-treated parasites showed reduced signal intensity consistent with developmental arrest at the ring-to-trophozoite transition. By 48 h, control parasites had reinvaded fresh erythrocytes showing a single ring-stage peak, whereas drug-treated populations displayed diminished and scattered signal patterns indicative of widespread parasite death following prolonged developmental arrest.

### Target validation by *in vitro* resistance evolution in *P. falciparum*

**Figure 2.**
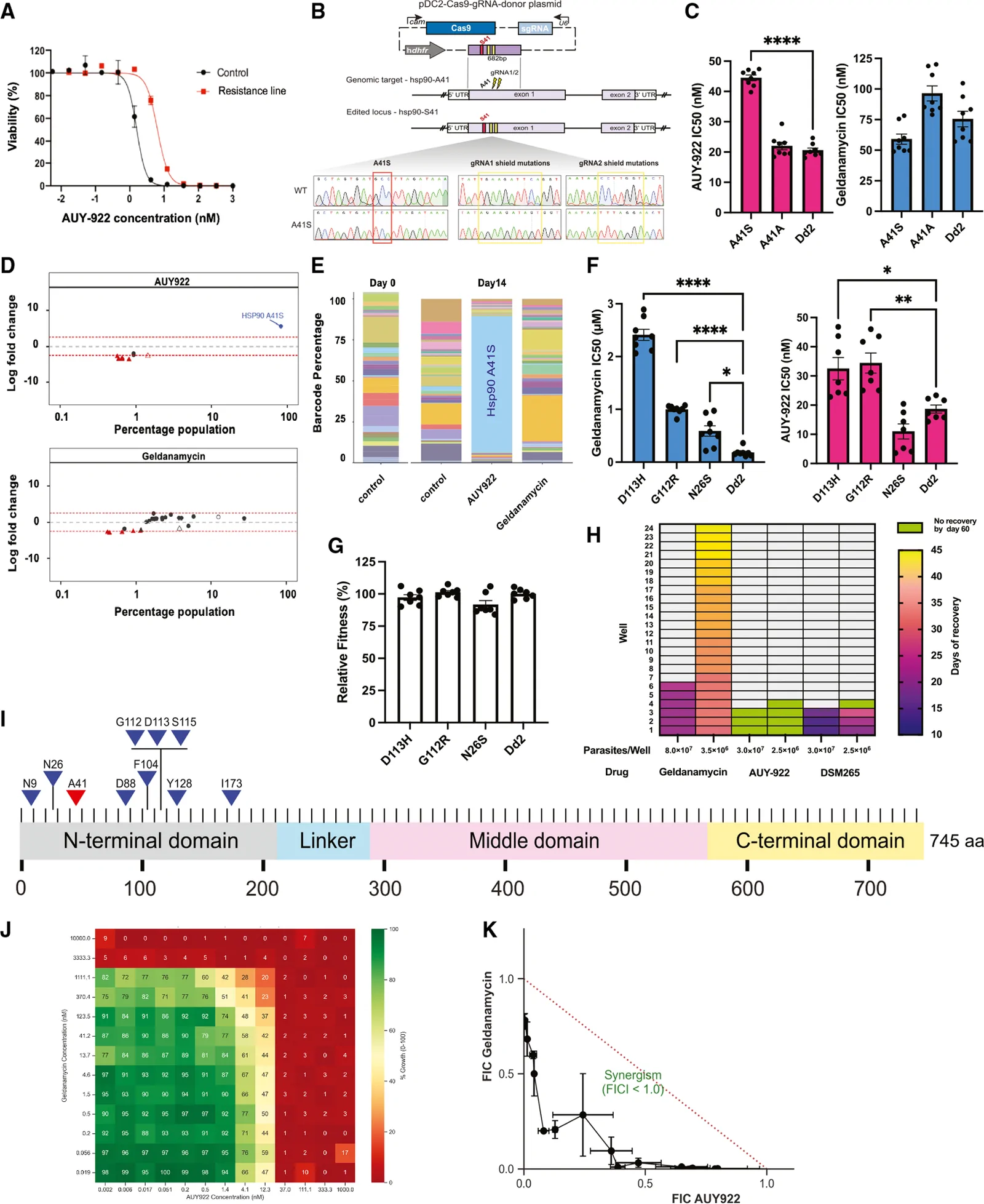
Resistance analysis and validation of HSP90 inhibitors (A) Dose-response curves of parental Dd2 and AUY-922 resistant line evolved over 44 weeks. (B) CRISPR-Cas9 strategy for introducing the A41S mutation into PfHSP90, with Sanger sequencing confirmation of successful editing (mutated codon highlighted in red box). (C) IC_50_ values of CRISPR-engineered parasites against AUY-922 (left) and geldanamycin (right). (D) AReBar competitive growth assay showing log_2_ fold-change analysis under AUY-922 (top) and geldanamycin (bottom) selection pressure. Each dot (circles: Dd2 background, triangles: 3D7 background) represents a barcoded mutant (filled) or wild type (empty) line, with parasites <0.1% not shown (see Table S3). (E) Barcode percentage composition at day 0 and day 14 after drug selection. Each bar represents the proportion of a barcoded line. (F) IC_50_ values of geldanamycin-resistant clones against geldanamycin (left) and AUY-922 (right). (G) Relative fitness assessment of geldanamycin-resistant clones. (H) Minimum inoculum for resistance (MIR) assay results displayed as a heatmap. Color gradient indicates days of recovery (10–45 days), lime indicates no recovery by day 60, and light gray indicates no data point. (I) Schematic representation of PfHSP90 domain organization showing resistance mutation distribution. AUY-922 resistance mutation A41S (red triangle) and geldanamycin resistance mutations (blue triangles). (J) Checkerboard combination assay showing percentage growth inhibition of *P. falciparum* across a matrix of AUY-922 and geldanamycin concentrations. (K) Isobologram analysis of AUY-922 and geldanamycin combination. Points falling below the dotted line indicate synergism (FICI <1.0). Data are represented as mean ± SEM. *p < 0.05, ***p < 0.001, ****p < 0.0001 by Brown-Forsythe and Welch ANOVA with Dunnett’s T3 multiple comparisons test (C), (F), and (G). See also Figure S2 and Tables S1, S2, and S3.

*In vitro* evolution experiments provide a powerful approach to investigate mechanisms of drug resistance and potential drug targets in an organismal context. When parasites acquire resistance-conferring mutations in a suspected target after compound exposure, it can provide valuable evidence supporting on-target activity, though resistance mutations can also arise in other pathways that affect drug efficacy. Previous evolution experiments from our lab showed that *Saccharomyces cerevisiae* (*S. cerevisiae*) readily acquired multiple resistance mutations to AUY-922, all located in the N-terminal ATP-binding domain of ScHSP90 (Table 1).^15^ Given the high conservation of this domain between species, we anticipated finding similar resistance patterns in *P. falciparum*. However, the malaria parasite exhibited different behavior. Despite subjecting *P. falciparum* to an exceptionally prolonged selection period of 44 weeks with gradually increasing AUY-922 concentrations, resistance development proved remarkably difficult. This extensive evolution experiment yielded only a single resistant clone showing a 4-fold increase in IC_50_ (Figure 2A). Whole-genome sequencing identified an A41S amino acid change in the ATP-binding domain of PfHSP90, along with two additional mutations: a variant in the 5′ UTR of Pf3D7_0930500 and a missense mutation S1050F in Pf3D7_0916200 (Table S1). The nearly year-long selection pressure required to obtain even a single resistant clone in *P. falciparum* indicates an exceptionally high barrier to AUY-922 resistance in the malaria parasite compared to yeast. To determine whether A41S alone was sufficient to confer resistance, we introduced this mutation, or alternatively a silent A41A control mutation, into a clean genetic background using CRISPR-Cas9 editing (Figure 2B). Edited parasites were clonally selected and validated by Sanger sequencing, and subsequently assessed for AUY-922 sensitivity. These data showed that A41S but not the silent control mutation conferred a statistically significant 2-fold shift in IC50 to AUY-922 (Figure 2C), demonstrating that A41S is sufficient to confer the observed resistance phenotype. Notably, the A41S mutation did not confer cross-resistance to GA; instead, it rendered parasites modestly more sensitive to GA, further suggesting distinct resistance mechanisms for these two HSP90 inhibitors despite their shared binding site.

To address the potential contribution of the two additional mutations identified by whole-genome sequencing, we note that the Pf3D7_0930500 variant falls in a noncoding region, and as we have previously shown, noncoding single nucleotide variants (SNVs) rarely confer resistance phenotypes.^16^ Furthermore, the highly AT-rich nature of noncoding regions in *P. falciparum* severely limits the availability of suitable PAM sites, making CRISPR-Cas9 validation of this variant impractical. For Pf3D7_0916200 S1050F, CRISPR-Cas9 editing attempts were unsuccessful in recovering parasites carrying the mutant allele. Although editing was confirmed by the recovery of a silent mutation at the gRNA binding site, no parasites with the S1050F substitution could be obtained. Together, these data support A41S as the primary resistance determinant, though we acknowledge that the contribution of the additional mutations cannot be formally excluded.

To further validate the specificity of AUY-922 for HSP90 and test whether the compound also hits other known antimalarial targets, we employed the Antimalarial Resistome Barcode (AReBar) platform, which allows simultaneous tracking of a diverse panel of resistance mutations in a mixed population.^17^ In this system, the pool of parasite lines encompasses >30 different modes of action, with various validated resistance mutations to different antimalarial compounds, including mutations conferring resistance to artemisinin, atovaquone, and other clinical, preclinical and experimental antimalarials, including with our HSP90 A41S mutant (Table S3). Barcode frequency analysis of mixed populations under drug pressure (3 × IC_50_ for 14 days) revealed highly specific drug-resistance interactions (Figure 2D). The differential analysis established HSP90 A41S as the only variant showing significant enrichment (>2.5 log_2_ fold change) in AUY-922-treated cultures, corresponding to parasites harboring the HSP90 A41S mutation reaching over 90% of the population (Figure 2E), whereas all other resistance mutations were not appreciably enriched. In contrast, GA-treated populations showed no significant enrichment of the A41S variant, further confirming the lack of cross-resistance between these compounds (Figures 2D and 2E). Notably, the MCP (Mitochondrial carrier protein, PF3D7_0908800) P214T line showed increased representation in GA-treated cultures; however, this line is among the fittest in the pool and showed comparable enrichment in the no-drug control condition, indicating that its apparent enrichment reflects a general fitness advantage rather than specific GA resistance. Dose-response assays confirmed no significant difference in GA sensitivity between the MCP P214T and parental lines (Figure S2E).

We also examined GA resistance in malaria parasites using an early cloning protocol in which parasites are exposed to GA and then rapidly isolated before a clone would have an opportunity to grow out and outcompete everything else.^18^ In contrast to AUY-922, multiple GA-resistant parasites were readily obtained, with clones all showing increased IC_50_ values. Targeted HSP90 sequencing of six of these clones confirmed that all had acquired mutations conferring resistance to GA in the *P. falciparum* HSP90 gene. The identified mutations, found in separate clones, included N9K, D88Y, G112W, N26S, G112R, and D113H (Table 1). These resistant clones exhibited increased IC_50_ values for GA, with fold changes ranging from 1.8 to 22.3 compared to the wild-type Dd2 parasite. A key finding is the D88Y mutation, which occurs at a position analogous to E88 in *S. cerevisiae* Hsp82, previously identified as a resistance site for GA derivatives.^19^ In both organisms, mutations at position 88 replace an acidic residue (aspartic acid in PfHSP90, glutamic acid in yeast) with a non-acidic one, suggesting a conserved mechanism of resistance through altered drug-protein interactions in the ATP binding pocket.

### Cross-resistance testing reveals different mechanisms of action for HSP90 inhibitors

To further characterize the resistance mechanisms, we conducted cross-resistance profiling. These GA-resistant clones showed minimal cross-resistance to both AUY-922 (Figure 2F) and radicicol (Figure S2A). The log2 fold-change analysis revealed distinct resistance patterns: while all three tested mutations (D113H, G112R, and N26S) showed strong resistance to GA (positive log2 fold changes), they exhibited minimal or no resistance to AUY-922 and radicicol, with some mutations even showing slight sensitization (negative log2 fold changes) to these compounds (Figure S2B). To validate the specificity of these resistance patterns, we tested the GA-resistant clones against artemisinin and GNF179, antimalarial compounds with distinct mechanisms of action.^20,21^ As expected, these clones maintained wild-type sensitivity to both control compounds (Figures S2C and S2D), confirming that the resistance was specific to GA and did not reflect general drug resistance mechanisms. Furthermore, we assessed the fitness costs of these resistance mutations by comparing the growth rates of GA-resistant clones to wild-type parasites under normal culture conditions. No significant differences in growth rates were observed (Figure 2G), indicating that the GA resistance mutations do not impose a substantial fitness cost under these conditions.

### Comparative MIR analysis reveals differential resistance barriers

The differences between the evolution time and number of recovered resistant clones for GA and AUY-922 were surprising given that both compounds should target HSP90. To further quantify the propensity for resistance development, we conducted minimum inoculum for resistance (MIR) assays for both AUY-922 and GA (Figures 2H and S2F). This approach provides a quantitative measure of the likelihood of resistance development for each compound.^22^ For AUY-922, no resistant parasites were observed in any of the wells throughout the 60-days period. This result indicates an MIR value of at least 10^8^, supporting a high barrier to resistance for AUY-922. In contrast, GA-treated cultures exhibited resistant parasites between days 21 and 45, with a minimum inoculum of 3.5 × 10^6^ parasites required for the development of resistance. This lower MIR value indicates a higher propensity for resistance development compared to AUY-922, consistent with our earlier observations from the *in vitro* evolution experiments. The control compound, DSM265, showed resistant parasites between days 13 and 27, corresponding to an MIR value of ~3 × 10^6^. This result is consistent with its known propensity for resistance development.^23^ Sequencing analysis of the resistant parasites from the GA MIR assay revealed four additional mutations in PfHSP90: F104Y, S115Y, Y128H, and I173F (Table 1). These new mutations, combined with those identified in our earlier experiments, highlight the diverse ways in which *P. falciparum* can develop resistance to GA, all involving modifications to PfHSP90.

### AUY-922 and GA act synergistically in combination

Our evolution experiments yielded multiple resistance mutations across both compounds, and remarkably, all resistance-conferring mutations mapped exclusively to the N-terminal ATP-binding domain of PfHSP90 (Figure 2I). These resistance mutations include the single AUY-922 resistance mutation (A41S, shown in red) and multiple GA resistance mutations (N9K, D88Y, G112W, N26S, G112R, D113H, F104Y, S115Y, Y128H, and I173F, shown in blue). All mutations are located within the N-terminal ATP-binding domain near the expected binding site for GA based on crystal structures from other species.^24,25^ The exclusive localization of all resistance mutations within this conserved domain strongly supports HSP90 as the primary target for both compounds in *P. falciparum* and confirms that both inhibitors function through direct competition with ATP binding, consistent with their known mechanism of action in other organisms.

Given that both AUY-922 and GA target the same ATP-binding domain of PfHSP90, we next asked whether the two compounds interact synergistically, additively, or antagonistically when used in combination. We performed a checkerboard assay across a matrix of AUY-922 and GA concentrations and analyzed the interaction using isobologram analysis (Figures 2J and 2K). The fractional inhibitory concentration index (FICI) values fell below 1.0, indicating synergism between the two compounds. This result is somewhat unexpected, as two compounds competing for the exact same binding site would typically be predicted to show antagonism or additivity rather than synergism and suggests that despite sharing the same ATP-binding pocket, AUY-922 and GA may engage the target in subtly different ways or exert complementary effects on HSP90 function. These findings also suggest that combining AUY-922 and GA may represent a promising strategy to increase the barrier to resistance.

### Yeast model confirms HSP90 as target for GA and AUY-922

To further investigate the conservation of these resistance mutations, we used a yeast model. We systematically examined the functional impact of key mutations using CRISPR-based gene editing in yeast HSP90 (HSC82). After optimizing CRISPR-Cas9 vectors for the ABC16-Monster strain, which lacks 16 known adenosine triphosphate-binding cassette (ABC) transporters associated with drug resistance,^26^ we generated four mutations: A41S (the *P. falciparum* AUY-922 resistance mutation), E88Y (corresponding to the *P. falciparum* D88Y GA resistance mutation), A122P (identified in yeast AUY-922 evolution and equivalent to *P. falciparum* G112R GA resistance), and T171N (from yeast AUY-922 evolution).

**Figure 3.**
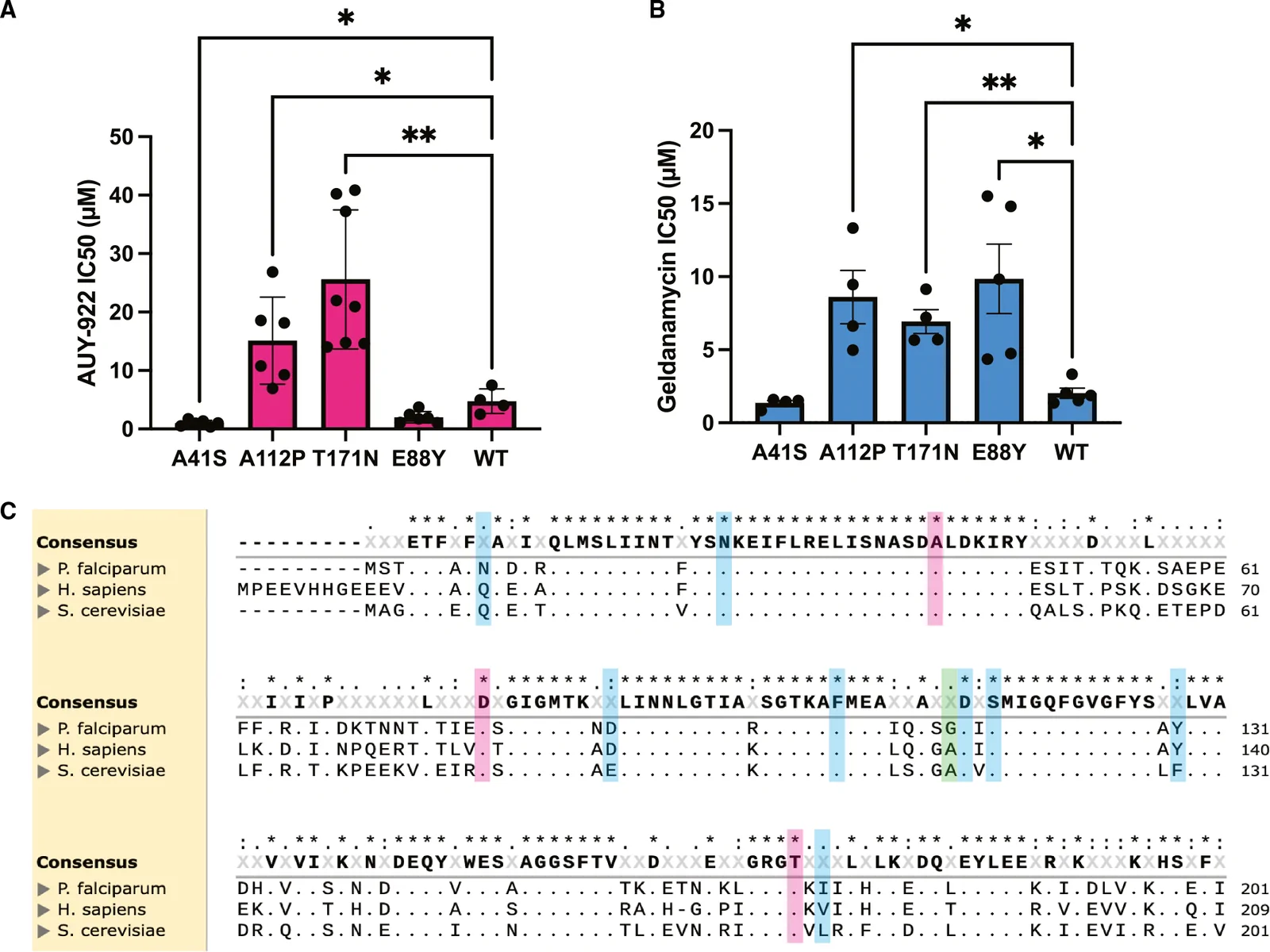
Cross-species comparison of HSP90 inhibitor sensitivity and resistance patterns (A) Cross-resistance analysis of CRISPR-engineered *S. cerevisiae* HSP90 mutations showing IC_50_ fold-changes against AUY-922. (B) Cross-resistance analysis of CRISPR-engineered *S. cerevisiae* HSP90 mutations showing IC_50_ fold-changes against geldanamycin. WT represents the wild-type ABC16-Monster strain. (C) Multiple sequence alignment of *P. falciparum* HSP90, *S. cerevisiae* HSC82, and human HSP90α. Resistance mutations identified from both *P. falciparum* and *S. cerevisiae* are highlighted: AUY-922 (red), geldanamycin (blue), and shared mutations (green), demonstrating conservation of key residues across species. Data are represented as mean ± SEM. *p < 0.05, ***p < 0.001, ****p < 0.0001 by Brown-Forsythe and Welch ANOVA with Dunnett’s T3 multiple comparisons test (A) and (B). See also Figure S3.

The engineered yeast strains revealed definitive and striking differences in their drug responses. Testing against AUY-922 (Figure 3A) showed that the A41S mutation produced opposite effects between species—while it confers AUY-922 resistance in *P. falciparum*, it dramatically increased sensitivity in yeast. The A122P and T171N mutations both conferred strong resistance to AUY-922, while E88Y showed increased sensitivity. Against GA (Figure 3B), E88Y demonstrated clear resistance, while A122P and T171N also showed resistance. The A41S mutation again showed increased sensitivity to GA in yeast. Cross-resistance testing with radicicol revealed similar patterns (Figure S3A), with A122P and T171N conferring resistance while A41S and E88Y showed sensitization. The comprehensive fold-change analysis (Figure S3B) confirmed these distinct response patterns across all three HSP90 inhibitors, clearly demonstrating fundamental differences in HSP90 inhibitor mechanisms between yeast and *P. falciparum*.

The nucleotide binding domain for HSP90 is highly conserved, showing 70% identity between *S. cerevisiae* Hsc82 and *P. falciparum* HSP90, through the first 211 residues, with similar conservation observed with human HSP90α (Figure 3C). Notably, AUY-922 resistance mutations are located at more highly conserved positions compared to GA resistance mutations.

### Structural characterization of resistance mutations and drug-target interactions

**Figure 4.**
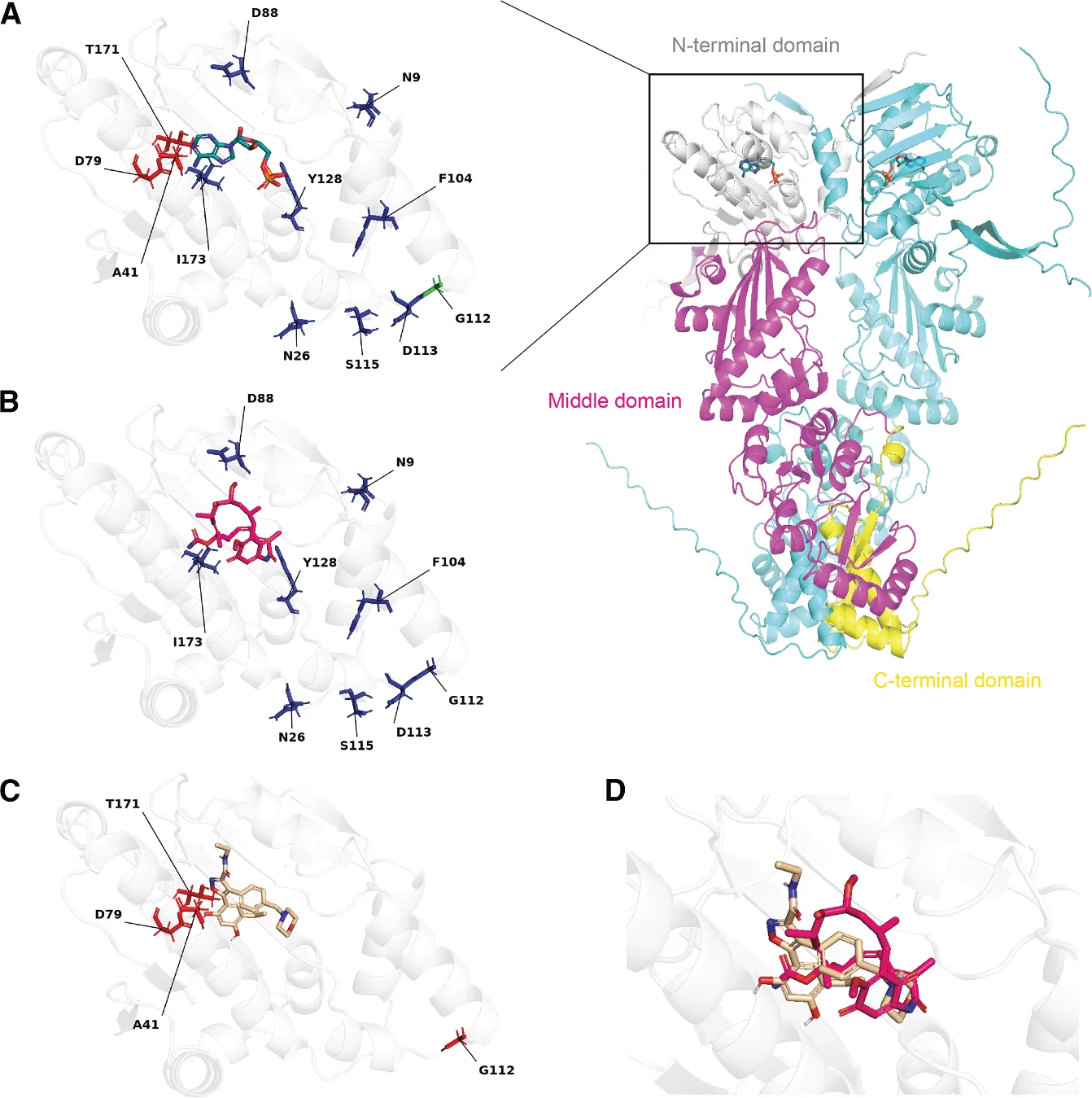
Structural analysis of resistance mutations and ligand interactions (A) Location of resistance mutations in PfHSP90 structure. Left: crystal structure (3K60) with ADP (turquoise) bound, showing resistance mutations from both *P. falciparum* and *S. cerevisiae* mapped onto the ATP-binding pocket. AUY-922 resistance mutations (red), geldanamycin resistance mutations (blue), and mutations conferring resistance to both inhibitors (green) are indicated. Right: full-length PfHSP90 homodimer structure in complex with ADP generated using AlphaFold3, showing overall protein architecture with the N-terminal domain (gray), middle domain (magenta), and C-terminal domain (yellow) of one monomer, and the second monomer in cyan. (B) Structural context of geldanamycin (magenta) resistance mutations mapped onto the PfHSP90 structure, showing their distribution throughout the N-terminal domain relative to the inhibitor binding site. (C) AUY-922 (tan) binding mode in PfHSP90 obtained through computational docking, showing the inhibitor positioned within the ATP-binding pocket. (D) Structural overlay demonstrating that both AUY-922 and geldanamycin bind to the same ATP-binding pocket of PfHSP90, with both inhibitors shown in the binding site to illustrate their overlapping binding regions despite different resistance profiles. See also Figure S4.

To understand the structural basis of resistance, we performed in silico analysis of the identified mutations using structural models of PfHSP90. The full-length PfHSP90 structure in complex with ADP was generated using AlphaFold3,^27^ showing the overall protein architecture with the N-terminal ATP-binding domain (Figure 4A). The left image shows the crystal structure (3K60) with resistance mutations from both *P. falciparum* and yeast mapped onto the ATP-binding pocket, revealing their spatial distribution relative to the active site.^28^ Notably, AUY-922 resistance mutations are located in close proximity to the ATP-binding pocket, while GA resistance mutations are positioned farther from the active site.

For detailed ligand-protein interaction analysis, we employed computational approaches to examine natural substrate and inhibitor binding. ADP binding to PfHSP90 was analyzed using PoseEdit,^29^ revealing the extensive interaction network formed by the natural substrate (Figure S4A). ADP forms critical hydrogen bonds with key residues including D79, N37, and F124, establishing the baseline interaction pattern for the ATP-binding pocket. Geldanamycin interactions were modeled through structural alignment with the yeast HSP90-GA complex, showing that resistance mutations are distributed throughout the N-terminal domain (Figure 4B). PoseEdit analysis revealed the detailed binding interactions of GA, though this analysis does not include resistance mutations (Figure S4B). Since most GA resistance mutations are located away from direct contact sites with the inhibitor, they likely affect binding through indirect mechanisms such as altering pocket conformation or dynamics. AUY-922 binding interactions were analyzed through computational docking followed by PoseEdit analysis (Figures 4C and S4C). The analysis revealed that AUY-922 resistance mutations are positioned near the bound inhibitor.

Structural overlay analysis demonstrated that both AUY-922 and GA bind to the same ATP-binding pocket of PfHSP90, confirming their shared target site despite their different resistance profiles (Figure 4D). This analysis revealed the spatial relationship between the two inhibitors within the binding pocket and provided insights into how their different binding modes may contribute to distinct resistance mechanisms. These structural insights provide a molecular basis for understanding the different resistance profiles observed for these compounds, with AUY-922 resistance mutations directly impacting both inhibitor binding and essential ATP/ADP interactions, explaining the higher barrier to resistance development. These findings align with the substrate envelope hypothesis, which posits that inhibitors mimicking the natural substrate binding mode can minimize the probability of resistance.^30^

### Binding affinity analysis reveals enhanced AUY-922 interactions with PfHSP90 A41S mutant

To validate the binding affinity differences between wild-type and mutant PfHSP90, we expressed and purified the N-terminal domains of PfHSP90 wild-type, PfHSP90 A41S, and PfGRP94 in *E. coli* using Ni-NTA chromatography. Protein purity was confirmed by SDS-PAGE and western blot analysis (Figure S5A), demonstrating successful expression and purification of all three proteins.

**Figure 5.**
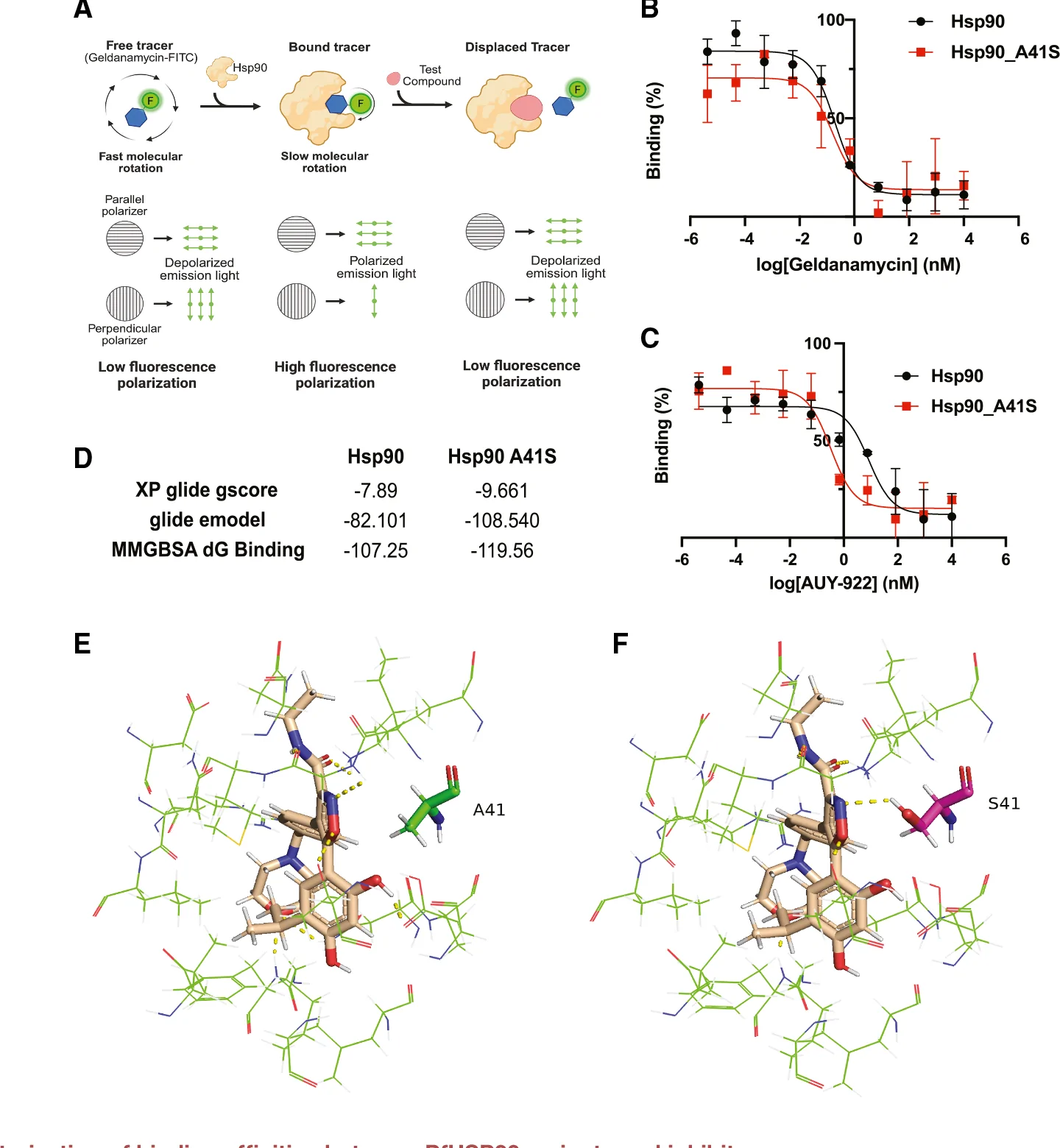
Characterization of binding affinities between PfHSP90 variants and inhibitors (A) Schematic of fluorescence polarization assay principle showing free, bound, and displaced GA-FITC tracer states, illustrating how competitive binding reduces polarization in a concentration-dependent manner. (B) Competitive binding curves for geldanamycin showing similar binding profiles between wild-type PfHSP90 (black) and A41S mutant (red), indicating unchanged binding affinity. (C) AUY-922 competitive binding curves demonstrating enhanced binding affinity for PfHSP90 A41S mutant (red) compared to wild-type PfHSP90 (black). (D) Computational binding analysis showing improved docking scores and binding energies for the A41S mutant across multiple metrics (XP Glide gscore, Glide emodel, and MM-GBSA). (E) Structural model of wild-type PfHSP90 with AUY-922 showing the A41 residue (green) position relative to the bound inhibitor. (F) Structural model of PfHSP90 A41S mutant with AUY-922 highlighting the S41 residue (magenta) and the additional hydrogen bonding opportunity (yellow dashed line) it provides. Data are represented as mean ± SEM of biological replicates. See also Figure S5.

We employed fluorescence polarization (FP) to measure competitive binding affinities. This technique exploits the principle that when a fluorescently labeled ligand binds to a target protein, its rotational diffusion becomes restricted, resulting in increased polarization of the emitted light (Figure 5A). We used fluorescein isothiocyanate (FITC)-labeled GA-FITC as the tracer, and the magnitude of polarization change directly reflects the fraction of bound ligand, providing a sensitive assessment of binding affinity. In competitive binding assays, unlabeled inhibitors (GA or AUY-922) competed with GA-FITC for binding to PfHPS90, causing concentration-dependent reductions in polarization that were fitted to derive Ki values.^31^

Our results revealed striking differences in binding behavior between the compounds and protein variants. For GA competition, binding affinity remained essentially unchanged between wild-type PfHSP90 and the A41S variant (Figure 5B), consistent with our cellular IC_50_ data showing that A41S does not confer resistance to GA. Note that PfGRP94 showed no detectable binding with GA-FITC under our assay conditions, precluding competitive binding analysis for this protein. In contrast, AUY-922 competition revealed enhanced binding affinity for the A41S variant compared to wild-type protein (Figure 5C). This finding is particularly intriguing because the A41S mutation, which confers resistance to AUY-922 in live parasites, actually increases drug binding affinity—contrary to traditional resistance mutations that typically decrease drug-target interactions. Computational analysis strongly supported this biochemical finding (Figure 5D). All computational metrics—XP Glide gscore,^32^ Glide emodel, and MM-GBSA binding free energy^33^—consistently demonstrated enhanced binding interactions for the A41S mutant compared to wild-type protein, corroborating the experimental FP results. Structural analysis revealed the molecular basis for this enhanced binding: while the wild-type A41 residue shows no direct hydrogen bonding with AUY-922 (Figures 5E and S4C), the A41S mutant provided a strong improvement in docking interactions (Figures 5F and S4D). The S41 residue contributed new hydrogen bonding interactions that were absent in the wild-type protein. This paradoxical enhancement of binding affinity in a resistant mutant represents a fundamentally different resistance mechanism compared to traditional mutations that reduce drug binding affinity.

To further investigate the effect of A41S on drug binding across organisms, we also expressed and purified the N-terminal domain of yeast HSC82 carrying the A41S mutation and performed FP competition assays (Figures S5B and S5C). Consistent with the result observed in *P. falciparum*, GA binding was unaffected by the A41S substitution in yeast HSC82. In contrast, A41S reduced AUY-922 binding affinity in yeast HSC82, as evidenced by a rightward shift in the competition curve relative to wild type, opposite to the enhanced binding observed in PfHSP90 A41S. This divergence in AUY-922 binding direction between the two organisms is consistent with their divergent phenotypic outcomes, where A41S confers AUY-922 resistance in *P. falciparum* but hypersensitivity in yeast. These findings highlight how the same amino acid substitution can produce fundamentally different biochemical and phenotypic consequences depending on the organism and its HSP90 isoform complement.

### HSP90 knockdown analysis reveals contrasting drug mechanisms

**Figure 6.**
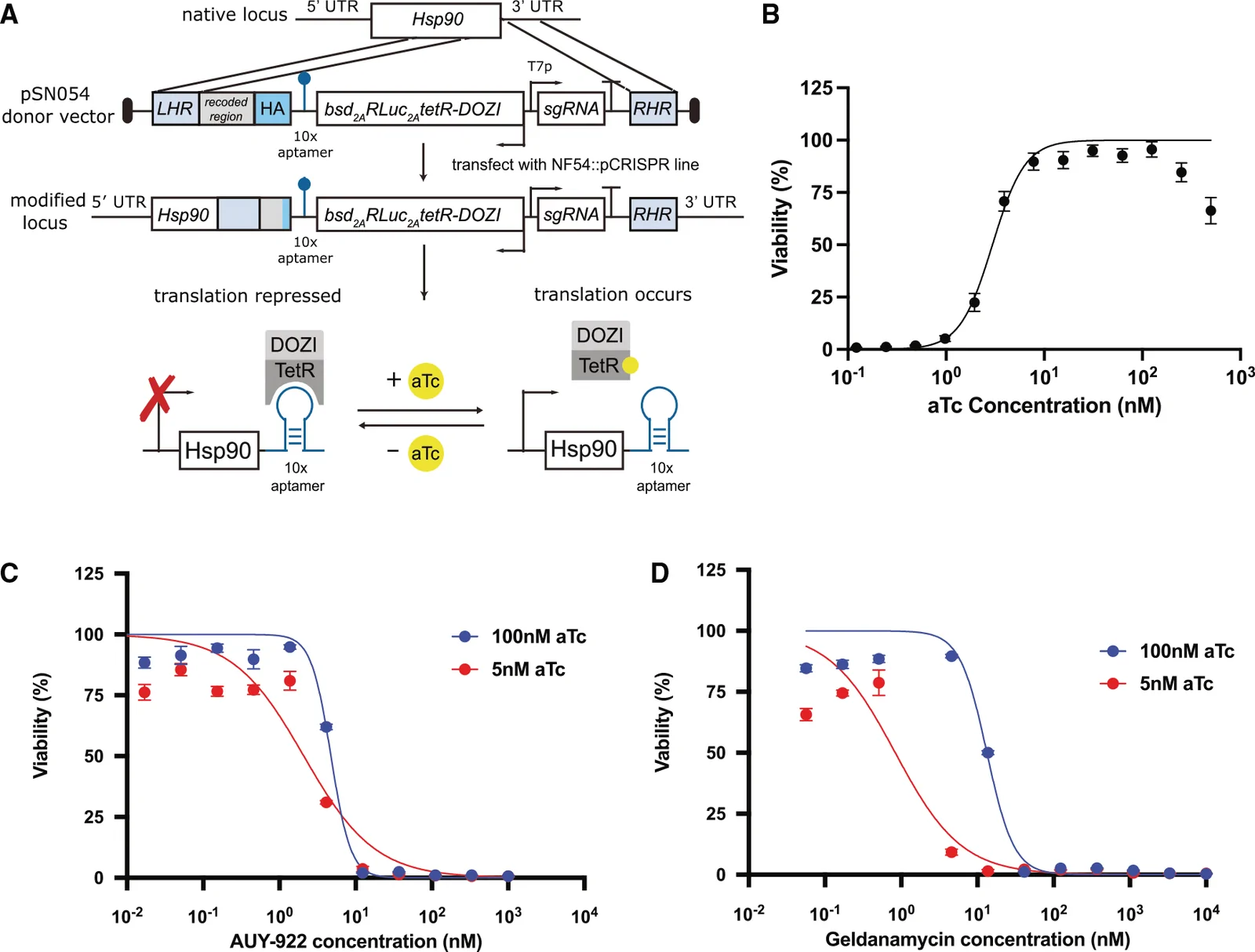
HSP90 conditional knockdown reveals differential dependency of inhibitor activity (A) Schematic representation of the PfDOZI-TetR conditional knockdown system, showing the integration of TetR-binding aptamer sequences, HSP90 epitope tag, PfDOZI-TetR expression cassette, and selection marker. (B) Dose-dependent regulation of HSP90 protein levels by varying concentrations of anhydrotetracycline (aTc). Optimization to achieve partial protein depletion while maintaining parasite viability. (C) IC_50_ shift assay for AUY-922 under HSP90 knockdown conditions comparing 5 nM vs. 100 nM aTc treatment. (D) IC_50_ shift assay for geldanamycin under HSP90 knockdown conditions comparing 5 nM vs. 100 nM aTc treatment. Data are represented as mean ± SEM of biological replicates. See also Figure S6.

To further understand the role of HSP90 as a drug target, we performed conditional knockdowns of HSP90 using the PfDOZI-TetR system previously described.^34,35^ In this system, PfDOZI-TetR binds to TetR-binding aptamer sequences incorporated downstream of the gene of interest, resulting in translational repression. This repression is relieved by adding anhydrotetracycline (aTc), which prevents PfDOZI-TetR from binding to the aptamers. Ideally, epitope tags would be incorporated in-frame with the PfHSP90 coding sequence to allow protein quantification by western blot; however, attempts to generate parasites without an intervening stop codon were unsuccessful, suggesting that the C terminus of PfHSP90 may be functionally important, and that direct addition of epitope tags at this position is not well-tolerated by the parasite. We therefore engineered a construct in which a stop codon was introduced immediately after the PfHSP90 coding sequence, followed by epitope tags and the 10× aptamer array, along with a PfDOZI-TetR expression cassette and a blasticidin-selectable (bsd) marker (Figure 6A). Using CRISPR-Cas9, we integrated this construct into the *P. falciparum* genome and confirmed successful integration by sequencing, which verified the presence of the introduced stop codon and the 10× aptamer array (Figure S6A). Given HSP90’s essential role in parasite survival, we carefully calibrated the system to achieve partial protein depletion without catastrophic effects on parasite viability. We determined that an aTc concentration of 5 nM provided an optimal balance (Figure 6B), allowing us to examine drug-target interactions while maintaining sufficient HSP90 function for parasite growth.

Under these conditions, we tested our conditional parasites against HSP90 inhibitors and control compounds. The results revealed striking differences between the compounds. AUY-922 showed almost no IC_50_ shift under HSP90 depletion conditions (Figure 6C), with similar IC_50_ values at both 100 and 5 nM aTc concentrations. In contrast, the conditional knockdown rendered parasites dramatically more sensitive to GA (Figure 6D), with a substantial leftward shift in the dose-response curve under HSP90 depletion (5 nM aTc) compared to the control condition (100 nM aTc). Similar patterns were observed with radicicol, another HSP90 inhibitor, which showed increased sensitivity under HSP90 knockdown conditions (Figure S6B). As a specificity control, we tested GNF179, which acts through an unrelated mechanism, and observed minimal difference between the two aTc conditions (Figure S6C), confirming that the differential effects were specific to HSP90 inhibitors. These data strongly suggest that AUY-922 may exert its antimalarial activity through a different mechanism than direct HSP90 inhibition, while confirming HSP90 as the primary target for GA and radicicol.

## DISCUSSION

Our findings reveal that AUY-922 exhibits an exceptionally high barrier to resistance development, representing a rare example of a highly resilient antimalarial compound. The stark contrast with GA—despite both targeting the same HSP90 binding site—challenges assumptions that compounds sharing identical targets exhibit similar resistance profiles. The most striking discovery is the paradoxical enhancement of AUY-922 binding by the single, recovered A41S mutation. We hypothesize that this mutation may create a molecular trap that sequesters drug away from critical cellular targets, rather than following the classical competitive inhibition pattern that typically weakens drug-target interactions. This enhanced binding mechanism may represent an underexplored paradigm in antimalarial drug development. We propose that A41S increases AUY-922 affinity for cytosolic PfHSP90 while reducing availability for other essential targets. This redistribution protects the critical members required for antiparasitic activity, conferring resistance despite stronger binding at the primary target. Mechanistically analogous phenomena have been observed in other systems where protein-mediated drug sequestration reduces effective target engagement and produces resistance—for example, in cancer cells, LAPTM4B overexpression sequesters doxorubicin and delays nuclear delivery,^36^ providing a precedent for protein-facilitated drug trapping as a resistance mechanism.

The differential isoform engagement of AUY-922 and GA is further supported by multiple lines of evidence. Our isobologram analysis revealed synergistic interactions between the two compounds (FICI <1.0), which is notable because two compounds competing directly for the same binding site would typically be expected to show antagonism or additivity rather than synergism. The observed synergism suggests that despite sharing the same ATP-binding pocket, AUY-922, and GA engage PfHSP90 family members in complementary ways, consistent with the differential isoform selectivity profiles of the two compounds and supporting the multi-target model. Our conditional knockdown experiments further reinforce this distinction, showing that HSP90 depletion minimally affected AUY-922 sensitivity but significantly increased sensitivity to GA and radicicol, pointing toward distinct mechanisms of action for these structurally different inhibitors despite their shared binding site. We propose a multi-target model where the antimalarial activity of AUY-922 depends on coordinated inhibition of multiple HSP90 family members, while GA acts primarily through cytosolic HSP90. This is further supported by previous surface plasmon resonance binding data demonstrating that AUY922 shows high affinity for both PfHSP90 and PfGRP94, while GA binds PfHSP90 but shows dramatically weaker affinity for PfGRP94.^7^ Together, these data support a multi-target model where the antimalarial activity of AUY-922 depends on coordinated inhibition of not only PfHSP90, while GA acts primarily through cytosolic HSP90.

The cross-species differences in A41S mutation effects further support this multi-target hypothesis. While A41S confers resistance in *P. falciparum* but increases sensitivity in *S. cerevisiae*, this opposing effect correlates with fundamental differences in HSP90 family complexity between the two organisms. *P. falciparum* expresses four HSP90 homologs across different cellular compartments—PfHSP90 (cytosolic), PfGRP94 (ER), PfTRAP1 (mitochondrial), and PfHSP90-A (apicoplast)—while *S. cerevisiae* has only two cytosolic variants: constitutively expressed HSC82 and stress-induced HSP82. The A41S mutation may redistribute AUY-922 binding in favor of cytosolic PfHSP90, thereby protecting other essential family members from inhibition in the more complex *P. falciparum* system. Among these potential protected targets, PfGRP94 has emerged as a promising antimalarial drug target, with recent studies demonstrating its druggability and essential roles in parasite protein export and survival.^37,38^

Emerging evidence highlights the attractiveness of developing “irresistible” antimalarial compounds that exhibit exceptionally high barriers to resistance.^39^ One potential mechanism for achieving such resistance barriers is through multi-target engagement. A compelling example of this principle is WM382, a dual plasmepsin IX/X antimalarial inhibitor that demonstrates an MIR value >10^9^ compared to single-target plasmepsin inhibitors, presumably because resistance would require simultaneous modifications to both target enzymes.^40^ Similarly, the vinyl sulfone WLL exhibits exceptionally low resistance propensity through dual targeting of proteasome β2 and β5 subunits.^41^ The exceptionally high MIR value observed for AUY-922 aligns with this principle, suggesting that effective antimalarial activity requires coordinated inhibition of multiple HSP90 family members.

Traditional drug discovery focuses on optimizing compounds against individual targets, but our findings suggest that deliberately designing compounds to engage multiple essential proteins within a family may provide superior resistance barriers. This principle aligns with broader polypharmacological strategies, where multi-target drugs have demonstrated superior resistance barriers across diverse therapeutic areas.^42^ This multi-target strategy extends beyond protein families to encompass different proteins within the same pathway, as exemplified by the antimalarial RYL-581, which simultaneously targets three distinct binding sites across separate mitochondrial respiratory chain proteins.^43^ However, this approach must be balanced against potential safety concerns. For infectious disease drugs, selectivity for pathogen targets over host proteins is crucial to minimize toxicity,^44–46^ and multi-target engagement could potentially increase the risk of host cell toxicity through broader effects on essential cellular proteins.

Our findings have important implications for antimalarial drug development. The contrasting resistance profiles of two compounds targeting the same binding site demonstrate that resistance risk cannot be predicted solely from target identity or binding mode. The identification of AUY-922’s resistance profile provides a framework for recognizing and developing compounds with enhanced durability against resistance. Understanding how to optimize compounds that resist evolutionary escape represents a fundamental advance in medicinal chemistry that could significantly extend the useful lifespan of therapeutic interventions, contributing to more sustainable malaria control strategies and broader efforts to combat drug resistance across diverse disease contexts.

### Limitations of the study

While this study provides mechanistic insights into the divergent resistance profiles of AUY-922 and GA in *P. falciparum*, several limitations should be acknowledged. First, although our CRISPR-Cas9 editing experiments demonstrate that A41S alone is sufficient to confer AUY-922 resistance, the potential contributions of the additional mutations identified by whole-genome sequencing, the Pf3D7_0930500 5′ UTR variant and Pf3D7_0916200 S1050F, cannot be formally excluded. Pf3D7_0916200 is a poorly characterized gene with limited functional literature and no ortholog assignments in standard databases. Based on Pfam domain prediction, it is tentatively annotated as having ribonuclease P activity, but its precise biological role in *P. falciparum* remains unclear. Second, the multi-target hypothesis proposing that AUY-922 engages multiple PfHSP90 family members requires direct experimental validation, as current evidence remains largely inferential. Direct binding measurements against PfGRP94 were precluded by the inability of the GA-FITC tracer to bind this isoform under our assay conditions. Future studies employing proteome-wide thermal proteome profiling or chemical proteomics with clickable derivatives would provide a more complete assessment of all protein targets engaged by AUY-922 and GA in the parasite.

## RESOURCE AVAILABILITY

### Lead contact

Requests for further information and resources should be directed to and will be fulfilled by the lead contact, Elizabeth A. Winzeler (ewinzeler@health.ucsd.edu).

### Materials availability

Selected *P. falciparum* resistant parasite lines and yeast strains carrying HSP90 mutations generated in this study are available from the lead contact with a completed materials transfer agreement.

### Data and code availability

- Whole-genome sequencing data from AUY-922-selected *P. falciparum* lines have been deposited at NCBI BioProject under accession SAMN37577935 (BioSample). Whole-genome sequencing data from GA-selected *P. falciparum* lines have been deposited at NCBI BioProject under accession PRJNA1430874, including parental and GA-selected bulk and clonal lines.
- This paper does not report original code.
- Any additional information required to reanalyze the data reported in this paper is available from the lead contact upon request.

## STAR★METHODS

### EXPERIMENTAL MODEL AND STUDY PARTICIPANT DETAILS

#### *Plasmodium falciparum* parasite lines

The *P. falciparum* Dd2 strain (chloroquine-resistant, MR4/BEI Resources MRA-156) and the Dd2-B2 clone derived from it were used for *in vitro* drug evolution and dose-response assays. The NF54 strain (MR4/BEI Resources MRA-1000), engineered to express Cas9 and T7 RNA polymerase,^35^ was used as the parental background for CRISPR-Cas9 editing of the A41S mutation and generation of the conditional HSP90 knockdown line.

#### Human blood donors

Human O+ whole blood (BioIVT, Hicksville, NY) was used for *P. falciparum* culture. Donors were screened and found negative for hepatitis B surface antigen, HBV NAT, HIV-1/2 antibody, HIV NAT, HCV antibody, HCV NAT, syphilis, West Nile virus NAT, and *T. cruzi* antibody, in accordance with FDA CBER-licensed screening tests (Title 21, CFR Part 610.40). Blood was obtained from commercial donors under BioIVT’s institutional ethical and regulatory donation standards. Donor sex, exact age (reported only as 40 years or younger), and other demographic information were not available to the researchers and were not considered in this study.

#### Anopheles stephensi mosquitoes and Plasmodium berghei sporozoites

*Plasmodium berghei* luciferase (PbLuc) sporozoites were obtained by dissection of salivary glands from infected *Anopheles stephensi* mosquitoes reared by The SporoCore, University of Georgia (Athens, GA, USA) under their institutional insectary protocols.

#### Human hepatocyte cell line

HepG2-A16-CD81-EGFP cells, a derivative of the human hepatocellular carcinoma cell line HepG2 (ATCC Cat#HB-8065), originally derived from a 15-year-old male donor, were used for liver-stage *P. berghei* infection and cytotoxicity assays.

#### Saccharomyces cerevisiae strains

The haploid *S. cerevisiae* ABC16-Monster strain (genotype: *lyp1Δ his3Δ1 leu2Δ0 ura3Δ0 met15Δ0*, MATa), in which all 16 ABC transporters implicated in multidrug resistance have been deleted^26^ was used as the parental background for CRISPR-Cas9 editing of HSC82 point mutations.

### METHOD DETAILSDETAILS

#### *P. falciparum* culturing

Parasites were cultured under standard conditions using RPMI media supplemented with 0.05 mg/mL gentamycin, 0.014 mg/mL hypoxanthine (prepared fresh), 38.4 mM HEPES, 0.2% sodium bicarbonate, 3.4 mM sodium hydroxide, 0.05% O+ human serum (denatured at 56°C for 40 min), and 0.0025% albumax. Leukocyte-free erythrocytes were stored at 50% hematocrit in RPMI-1640 screening media (as above, but without O+ human serum and with double albumax concentration) at 4°C for one to three weeks before experimental use. Cultures were monitored every one to two days via light microscopy-based observation of Giemsa-stained thin blood smears.

#### Drug preparation

The drug stock solutions were prepared by dissolving the compounds in dimethyl sulfoxide (DMSO) to achieve concentrations of 100 mM. Aliquots of 10 mM working stocks were prepared from these stock solutions and stored at −20°C for long-term storage. In all experiments, the final concentration of DMSO was maintained below 0.5% to minimize potential solvent effects.

#### *In vitro* drug sensitivity and IC_50_ determinations

*In vitro* drug susceptibility was determined by quantifying the total parasitic DNA content using SYBR Green staining in a 384-well plate format. Synchronized ring-stage *P. falciparum* Dd2-B2 clone parasites were cultured in the presence of 12-point, 2-fold serial dilutions of the test compounds in black, clear-bottom plates. An artemisinin dilution series was conducted in parallel as a positive control. After a 72-h incubation under standard culture conditions, the SYBR Green I fluorescent nucleic acid dye was added to the cultures. The fluorescence signal, which is proportional to the amount of parasitic DNA present, was measured using a Spectramax M5 plate reader (Molecular Devices) with excitation and emission wavelengths of 480 nm and 530 nm, respectively. Following positive control fluorescence subtraction and signal normalization, the IC50 values (effective concentration for 50% growth inhibition) were calculated using the Levenberg-Marquardt algorithm implemented in the Collaborative Drug Discovery database (Burlingame, CA. www.collaborativedrug.com).

#### Liver stage assay

Compounds were dissolved in DMSO (10 mM), and 10 nL of each compound (0.1% final DMSO concentration per well) was transferred using an ECHO 650 (Beckman) into the assay plates within the range of 10 μM to 0.5 nM atovaquone (1 μM) and 0.1% DMSO served as the positive and negative controls, respectively. Human hepatic cells (3 × 10^3^; HepG2-A16-CD81-EGFP) in 5 μL of DMEM medium (2 × 10^5^ cells/mL, 5% FBS, 5*X* Pen/Strep/Glu) were seeded in 1536-well plates (Greiner BioOne) 20 hours prior to the actual infection. PbLuc sporozoites were dissected from the salivary glands of infected Anopheles stephensi mosquitoes and filtered twice through a 20 μm nylon pore cell strainer.^13^ *Plasmodium berghei* luciferase (PbLuc) sporozoites were obtained by dissecting salivary glands of infected Anopheles stephensi mosquitoes provided by The SporoCore, University of Georgia, GA, USA (SporoCore.uga.edu). The sporozoites were resuspended in screening media, counted using a hemocytometer, and their final concentration was adjusted to 200 sporozoites per μL. The HepG2 cells were then infected with 1,000 sporozoites per well (5 μL), and the plates were spun down at 37°C for 3 min in an Eppendorf 5810 R centrifuge with a centrifugal force of 330 RCF on the lowest acceleration and brake settings. After incubation at 37°C in 5% CO_2_ for 48 h, the media was removed by spinning the inverted plates at 130 RCF for 1 min 2 μL of Bright-Glo Luciferase Assay System (Promega) was dispensed into the wells. Immediately after the addition of the Bright-Glo reagent, the plates were read by the Pherastar FSX reader (BMG Labtech). Luminescence intensity values were normalized against the positive (Atovaquone) and negative (DMSO) controls. All experiments were conducted in four technical replicates and repeated three times.

#### Cytotoxicity assay

Plates were prepared according to the liver stage conditions, except 5 μL of screening media was used instead of sporozoites.^13^ After incubation at 37°C for 48 h, the media was removed by spinning the inverted plates at 130 RCF for 1 min. Then, 2 μL of CellTiter-Glo Luminescent Cell Viability Assay (Promega) was dispensed into each well. Immediately after adding the CellTiter-Glo reagent, the plates were read using the Pherastar FSX reader (BMG Labtech). Luminescence intensity values were normalized against positive (Puromycin) and negative (DMSO) controls. IC_50_ values were calculated in CDD Vault (Burlingame, CA). All experiments were conducted in four technical replicates and repeated at least two times.

#### Giemsa staining and parasitemia quantification

To assess the morphological effects of HSP90 inhibitors on *P. falciparum*, tightly synchronized ring-stage parasites at 1% parasitemia were treated with 5× IC_50_ concentrations of AUY-922 or geldanamycin, or an equivalent volume of DMSO as a control. Thin blood smears were prepared at 24, 48, and 72 h post-treatment by spreading a small volume of parasite culture onto glass slides, which were then air-dried and fixed with 100% methanol. Slides were stained with 10% Giemsa solution (Sigma-Aldrich) for 5 min, rinsed with water, and air-dried. Parasite morphology and developmental stage were assessed by light microscopy at 100× magnification under oil immersion. Parasitemia was determined by counting the number of infected erythrocytes per 1000 total erythrocytes from each smear.

#### Determination of parasite stage by flow cytometry

*P. falciparum* cultures were first synchronized using 5% w/v sorbitol solution to obtain a population of ring-stage parasites. The synchronized cultures were then adjusted to 2% hematocrit and 2% parasitemia in T12.5 flasks. These cultures were treated with either 5 × IC_50_ of AUY-922, 5 × IC_50_ of geldanamycin, or an equivalent volume of DMSO as a control. The treated cultures were incubated for a total of 48 h under standard culture conditions. At 24- and 48-h post-treatment, samples were collected for flow cytometric analysis. The parasites were stained with 2μM SYTO61, a red fluorescent nucleic acid stain, for 20 min at room temperature. Following staining, the samples were washed with PBS to remove excess dye. Flow cytometry was then performed to analyze the DNA content and subsequently determine the distribution of parasite stages in each treatment condition.

#### *In vitro* resistance evolution in *Plasmodium*

Independent *P. falciparum* Dd2 clones were subjected to gradually increasing concentrations of AUY-922 over 44 weeks or geldanamycin over 8 weeks, starting at IC30 concentrations. The parasitemia levels were monitored daily by visual inspection using light microscopy of Giemsa-stained thin blood smears prepared from the parasite-infected erythrocyte cultures. Resistance was defined as achieving an IC50 value (the concentration for 50% growth inhibition) at least 2-fold higher than that of the untreated parental Dd2 strain. We also examined geldanamycin resistance using an early cloning protocol as previously described,^18^ where parasites were treated with 3× IC90 concentrations for 96 h, then seeded in 96-well plates and monitored for growth recovery. Upon attaining resistance phenotypes, genomic DNA was extracted from both the resistant cultures and the parental line for whole-genome sequencing analysis or PCR amplification of the PfHSP90 DNA sequence, which was then sent for Nanopore sequencing (Plasmidsaurus).

#### Minimum inoculum of resistance (MIR)

The MIR assay was performed using single-step continuous pressure of 3×IC_90_ drug concentrations for geldanamycin and 3.5×IC_50_ for AUY-922 and the DSM265 control on two parasite inoculum groups per compound. For AUY-922 and DSM265 control, inoculum sizes were 2.5 × 10^6^ parasites (four wells; 2% hematocrit) and 3 × 10^7^ parasites (three wells; 3% hematocrit). Geldanamycin testing used 3.5 × 10^6^ parasites per well in 24-well plates and 4.8 × 10^8^ parasites per w ell in 6-well plates. Wells were monitored daily by thin blood smear during the initial drug killing phase until parasite clearance. Geldanamycin cultures received additional monitoring by flow cytometry (24-well plates) and Giemsa-stained smears (6-well plates). Following clearance, drug media was changed three times weekly, cultures were passaged weekly by replacing one-third of the culture volume with fresh RBC media and monitored twice weekly by flow cytometry for recrudescence over 60 days. Genomic DNA from resistant cultures and parental lines was extracted for whole-genome sequencing analysis.

#### Whole-genome sequencing and analysis

Cultures were initially lysed with 0.15% saponin and washed twice with 1*x* PBS. Genomic DNA was extracted using the QIAamp DNA Blood Mini Kit (Qiagen) according to the manufacturer’s instructions. Whole-genome sequencing (WGS) libraries were prepared using the Nextera Flex DNA Library Preparation Kit (Illumina) following the manufacturer’s protocol. The libraries were multiplexed and sequenced on an Illumina MiSeq platform to generate 300 bp paired-end reads. The generated sequencing reads were quality-checked using FastQC (version 0.11.9) and trimmed using Trimmomatic (version 0.39) to remove adapters and low-quality bases. The trimmed reads were then aligned to the *P. falciparum* 3D7 reference genome (PlasmoDB, version 48) using the Burrows-Wheeler Aligner (BWA-MEM, version 0.7.17). PCR duplicates were removed using Picard (version 2.23.8), and unmapped reads were filtered out using SAMtools (version 1.10). Base quality score recalibration (BQSR) was performed using the Genome Analysis Toolkit (GATK, version 4.1.8) to adjust for systematic errors in base quality scores. The GATK HaplotypeCaller was then used to identify potential single nucleotide variants (SNVs) in the test parasite lines. The identified SNVs were filtered based on the following quality scores: variant quality as a function of depth (QD) > 1.5, mapping quality (MQ) > 40, minimum base quality score (MBQS) > 18, and read depth (DP) > 5. The resulting high-quality SNVs were annotated using SnpEff (version 4.3t) to predict their functional impact on genes and proteins.

#### CRISPR/Cas9 gene editing in *P. falciparum*

To validate the role of the A41S mutation in PfHSP90 in conferring resistance to AUY-922, we performed CRISPR/Cas9-mediated gene editing in the *P. falciparum* Dd2 strain. As a control, we also generated a synonymous mutation at the same position (A41A). The CRISPR/Cas9 plasmid, pDC2-coCas9-gRNA, containing a codon-optimized SpCas9 and a guide RNA (gRNA) cassette, was used for gene editing.^48^ The gRNA targeting the PfHSP90 locus was designed using Benchling (www.benchling.com) and cloned into the *Bbs*I site of the plasmid. The donor repair template, encoding the A41S mutation and silent shield mutations at the gRNA-binding site to prevent re-cutting, was synthesized as a double-stranded DNA fragment (GeneArt, Thermo Fisher Scientific) and cloned into the AatII/EcoRI sites of the plasmid using NEBuilder HiFi DNA Assembly (New England Biolabs). Successful insertion of the gRNA and donor template was confirmed by Sanger sequencing.

Synchronized ring-stage *P. falciparum* Dd2 parasites at 10% parasitemia were electroporated with 50 μg of the CRISPR/Cas9 plasmid. Parasites were resuspended in cytomix (120 mM KCl, 0.2 mM CaCl_2_, 2 mM EGTA, 10 mM MgCl_2_, 25 mM HEPES, 5 mM K2HPO4, 5 mM KH2PO4; pH 7.6) to a final volume of 420 μL and electroporated using a Bio-Rad Gene Pulser II (0.31 kV, 950 μF). After a 1-h recovery, the electroporated parasites were washed and cultured in complete medium at 3% hematocrit. Drug selection with 5 nM WR99210 (Jacobus Pharmaceuticals) was applied 24 h post-electroporation and maintained until day 8. Surviving parasites were then cultured without drug pressure, and clonal populations were obtained by limiting dilution. Genomic DNA was extracted from the clonal parasite lines using the QIAamp DNA Blood Mini Kit (Qiagen), and the PfHSP90 locus was PCR-amplified using primers flanking the edited region (Table S2). The presence of the desired mutations was confirmed by Sanger sequencing.

#### Antimalarial Resistome Barcode (AReBar) assay for target deconvolution

To investigate the impact of HSP90 inhibitors on different *P. falciparum* drug resistant lines (Table S3), we employed a competitive growth assay using 52 DNA barcoded lines covering >30 modes of action, essentially as previously described.^17^ The pooled culture was divided into three conditions: untreated control, AUY-922 at 3 × IC_50_, and geldanamycin at 3 × IC_50_. Cultures were maintained at 2% hematocrit and 1–5% parasitemia for 14–18 days, with media and compound replaced every 48 h. Samples were collected at day 0 and day 14, and the barcode region was amplified from saponin-lysed pellets using primers p1356 and p1357 (Table S2) for sequencing using the minION (Mk1d) platform. Sequencing data was processed to determine the relative proportion of each barcode over time, normalized to its initial proportion, and log2-transformed. Differentially represented barcodes under HSP90 inhibitor treatment compared to the untreated control were identified using the DESeq2 R package.^49^

#### CRISPR-Cas9 allelic replacements in *S. cerevisiae*

CRISPR/Cas9 vectors p414 and p426 from the Church lab (Addgene)^47^ were modified for compatibility with the ABC16-Monster strain. The Trp selection marker of p414 was replaced with met15, and the Ura selection marker of p426 was replaced with leu2. Cas9-expressing ABC16-Monster yeast cells were generated by transforming the met15-modified p414 plasmid using the standard lithium acetate method and selecting on CM-glucose plates lacking methionine. All DNA sequences were amplified using Q5 High-Fidelity DNA Polymerase (New England Biolabs) (Table S2). Donor DNA containing the desired allelic replacements was synthesized as gBlocks Gene Fragments (Integrated DNA Technologies). The p426 plasmid with the desired gRNA sequence was constructed using a three-fragment HiFi assembly. PCR products were treated with DpnI (New England Biolabs) at 37°C for 1 h, followed by heat inactivation at 80°C for 20 min. The assembly reaction was performed at 50°C for 30 min, and the resulting product was transformed into 5-alpha Competent E. coli (New England Biolabs). Cas9-expressing ABC16-Monster yeast cells were transformed with the gRNA plasmid and donor DNA fragments, and transformants were selected on CSM plates lacking methionine and leucine. Genomic DNA from single colonies was isolated, and the presence of the desired mutations was initially confirmed by Sanger sequencing (Eton Bioscience).

#### *S. cerevisiae* dose-response assays

To evaluate compound activity against yeast cells, single colonies of the ABC16-Monster strain harboring the desired mutations were inoculated into 3 mL of YPD media and cultured overnight at 30°C with shaking. The following day, cultures were diluted to log phase, and 50 μL of the diluted cultures were dispensed into the wells of a 384-well plate. Nine 1:2 serial dilutions of each compound were prepared in biological duplicates, resulting in final concentrations ranging from 0.005 to 100 μM. The plate was incubated at 30°C for 18 h, and the optical density at 600 nm (OD_600_) was measured. IC_50_ values were calculated by subtracting the OD_600_ values of positive controls (cells treated with 250 μM fluconazole) and performing nonlinear regression on [inhibitor] vs. response with a variable slope using GraphPad Prism. As a negative control, parallel cultures without compound treatment were included.

#### Computational modeling and docking analysis

The Schrodinger Maestro small molecule modeling suite was used for all modeling performed. The PfHSP90 crystal structure (PDB: 3K60, PF3D7_0708400) from the Protein DataBank was used for docking AUY-922 and modeling the *P. falciparum* A41S mutant.^28^ The Schrödinger Prime package was used to create the mutant residue and select the optimal sidechain conformation within the binding pocket.^50^ Prime minimization was performed to determine a low-energy protein conformation with minimal deviation from the starting X-ray coordinates. The N-terminal domain containing the ATP-binding pocket was isolated from each structure for docking analysis. Protein structures were prepared using the Protein Preparation Wizard and refined to alleviate steric clashes and structural strain.^51^ The AUY-922 ligand was prepared using LigPrep with default parameters.^52^ Glide XP (extra precision) docking was employed to dock AUY-922 into all three binding sites.^53^ The resulting docked poses were analyzed using the Prime MM-GBSA workflow to calculate binding free energies (ΔG) and estimate relative binding affinities.^54^ For geldanamycin analysis, we aligned the 3K60 structure with the yeast HSP90 structure in complex with GA (PDB: 1A4H). The full-length PfHSP90 structure in complex with ADP was generated using AlphaFold3. Final docked poses were exported to PyMOL for structural visualization, analysis, and figure preparation.

#### Protein construct and purification

The N-terminal domains of *P. falciparum* HSP90, GRP94 and yeast HSC82, each containing a C-terminal hexahistidine tag, were cloned into the pET21 vector and transformed into NiCo21(DE3) competent E. coli cells (New England Biolabs) (Table S2). Successful transformants were grown overnight at 37°C in LB medium supplemented with 100 μg/mL ampicillin. For protein expression, 200 mL LB cultures containing ampicillin were inoculated and grown at 37°C until OD reached 0.6. Protein expression was induced with 1 mM IPTG and cultures were incubated overnight at 16°C. Cells were harvested by centrifugation at 4,000 × g for 20 min at 4°C and resuspended in lysis buffer (5% glycerol, 0.3 M NaCl, 20 mM HEPES, 30 mM imidazole-HCl, 0.5% CHAPS, 21 mM MgCl_2_, 2% CHAPS, 0.1 mg/mL lysozyme, 0.5 U/mL benzonase, and Protease Inhibitor Cocktail). After sonication and clarification at 12,000 × g for 20 min, the supernatant was purified using Ni-NTA chromatography, washed extensively (5% glycerol, 0.3 M NaCl, 20 mM HEPES, 30 mM imidazole-HCl, and 21 mM MgCl_2_), and eluted with a stepwise imidazole gradient (150–350 mM). Purified proteins were concentrated using 10 kDa MWCO Amicon filters and buffer exchanged into 50 mM HEPES (pH 7.5), 500 mM NaCl. Protein purity was assessed by SDS-PAGE and concentrations were determined using the Qubit Protein Assay Kit.

#### Fluorescence polarization binding assay

Binding affinities between HSP90 inhibitors and purified PfHSP90 or PfHSP90-A41S were determined using FITC-labeled GA (GA-FITC) in a competition assay format. Initial direct binding experiments with serial dilutions of the purified proteins and GA-FITC in HSP90 assay buffer (BPS Bioscience) containing 2 mg/mL BSA and 2 mM DTT established optimal conditions. The Kd values from these experiments guided the selection of protein (10 nM) and GA-FITC (1 nM) concentrations for competition assays. Test compounds were serially diluted in DMSO starting from 1 mM with eight 10 × dilutions and added to the protein/GA-FITC mixture. Assays were performed in 384-well black, low-binding microplates with 50 μL final volume. After 16 h incubation at 4°C with gentle shaking, fluorescence polarization was measured using a PHERAstar FSX plate reader (excitation 485 nm, emission 530 nm).

#### Generation of conditional knockdown (cKD) parasite lines

To investigate the role of HSP90 as a drug target, we used CRISPR-Cas9 to generate parasite cell lines stably expressing the TetR-DOZI-RNA aptamer module for conditional regulation of HSP90 expression. These transgenic lines also contained the reporter construct *Renilla* luciferase (RLuc) and the selection marker BSD. To construct the donor plasmids, PCR-amplified right homology regions and synthesized DNA fragments corresponding to the left homology regions fused to the recodonized 3′-end of the HSP90 gene with 10× TetR-binding aptamers inserted at the 3′-end, as well as the target-specifying gRNA sequences, were cloned via Gibson assembly into the pSN054 linear vector. The donor vector enables fusion of 2*x* hemagglutinin tag to the protein, but this approach did not yield viable parasites, therefore the final edited cell line was not tagged. The final constructs were confirmed by restriction digests and Sanger sequencing.

Transfections into Cas9-and T7 RNA polymerase–expressing NF54 parasites were carried out by preloading erythrocytes with the donor plasmids as described previously.^55^ Cultures were maintained in 500 nM anhydrotetracycline (aTc; Sigma-Aldrich, 37919) and blasticidin-S (2.5 μg/mL; RPI Corp B12150–0.1). Parasite cell lines stably integrating the donor plasmids were monitored via Giemsa smears and RLuc measurements.

#### Conditional knockdown viability assays

To assess the effect of conditionally perturbing HSP90 expression on drug sensitivity, parasites were washed three times with media and then cultured in the presence of aTc (100 nM and 5 nM) at 2% hematocrit and 0.5% parasitemia for 72 h. Drug sensitivity assays were performed in triplicate using 384-well plates, and luminescence signals were measured using either PHERAstar FSX or a GloMax (Promega) plate reader following the Renilla-Glo Luciferase Assay System (Promega) manual.

### QUANTIFICATION AND STATISTICAL ANALYSIS

All statistical analyses were performed using GraphPad Prism software (version 10). For comparisons involving three or more groups with unequal variances, Brown-Forsythe and Welch ANOVA tests were performed, followed by Dunnett’s T3 multiple comparisons test. When comparing two groups, an unpaired *t* test with Welch’s correction was used. Statistical significance was defined as follows: *p < 0.05, **p < 0.01, ***p < 0.001, and ****p < 0.0001. All data are presented as mean ± SEM. Sample sizes (*n*) and the number of biological and technical replicates for each experiment are reported in the corresponding figure legends. All experiments were performed with at least three biological replicates and at least three technical replicates per biological replicate.

## Supplementary Material

1

2

3

4

SUPPLEMENTAL INFORMATION

Supplemental information can be found online at https://doi.org/10.1016/j.celrep.2026.117664.

## Figures and Tables

**Table 1.** Mutations identified in *in vitro* evolution or MIR experiments and IC_50_ fold-shift with AUY-922 and geldanamycin in *P. falciparum* and *S. cerevisiae*

AUY-922			Geldanamycin		
Nucleotide	Amino acid	IC_50_ shift- fold	Nucleotide	Amino acid	IC_50_ shift -fold

aaC/aaA	A41S	4.0	aaC/aaA	N9K	22.3
aCc/aAc ^a^	T171N ^a^	6.7^a^	Gat/Tat	D88Y	3.8
Gcc/Ccc ^a^	A112P ^a^	6.1^a^	GgA/TgG	G112W	1.8
gaT/gaG ^a^	D79G ^a^	37.8^a^	aAc/aGc	N26S	3.1
–	–	–	Gga/Cga	G112R	5.9
–	–	–	Gat/Cat	D113H	12.2
–	–	–	tTt/tAt	F104Y	5.0
–	–	–	tCt/tAt	S115Y	3.3
–	–	–	Tat/Cat	Y128H	2.6
–	–	–	Att/Ttt	I173F	5.3

a Yeast mutation (*S. cerevisiae* ABC16-Monster).

**KEY RESOURCES TABLE:** 

REAGENT or RESOURCE	SOURCE	IDENTIFIER
Antibodies		
Mouse anti-Histidine Tag antibody, clone AD1.1.10	Bio-Rad	Cat#MCA1396; RRID:AB_321842
Goat anti-Mouse IgG (H/L):HRP secondary antibody	Bio-Rad	Cat#STAR207P; RRID:AB_11125547
Chemicals, peptides, and recombinant proteins		
AUY-922 (Luminespib, NVP-AUY922)	MedChemExpress	Cat#HY-10215
Geldanamycin	MedChemExpress	Cat#HY-15230
Radicicol	MedChemExpress	Cat#HY-13251
Anhydrotetracycline (aTc)	Sigma-Aldrich	Cat#37919
Blasticidin-S	RPI Corp	Cat#B12150–0.1
Critical commercial assays		
HSP90α (N-Terminal) Geldanamycin Competitive Inhibitor Assay Kit (GA-FITC)	BPS Bioscience	Cat#50298
SYTO61 Red Fluorescent Nucleic Acid Stain	Thermo Fisher Scientific	Cat#S11343
SYBR Green I nucleic acid stain	Thermo Fisher Scientific	Cat#S7563
Renilla-Glo Luciferase Assay System	Promega	Cat#E2720
Qubit Protein Assay Kit	Thermo Fisher Scientific	Cat#Q33211
Deposited data		
Whole-genome sequencing data (AUY-922 selected P. falciparum lines)	NCBI BioSample	SAMN37577935
Whole-genome sequencing data (geldanamycin selected P. falciparum lines)	NCBI BioProject	PRJNA1430874
Experimental models: Cell lines		
Human: HepG2 hepatocytes	ATCC	Cat#HB-8065
Experimental models: Organisms/strains		
Plasmodium falciparum: NF54 strain	MR4/BEI Resources	MRA-1000
Plasmodium falciparum: Dd2B2 strain	Fidock lab	N/A
Plasmodium falciparum: PfHSP90 conditional knockdown line	This paper	N/A
Plasmodium falciparum: A41S CRISPR-edited line	This paper	N/A
Plasmodium berghei: luciferase reporter strain	Swann et al.^13^	N/A
Saccharomyces cerevisiae: ABC16-Monster strain (wild type)	Suzuki et al.^26^	N/A
Saccharomyces cerevisiae: HSC82 CRISPR-edited strains	This paper	N/A
Oligonucleotides		
Primers for CRISPR editing and protein expression, see Table S2	This paper	N/A
Recombinant DNA		
pET21-PfHSP90-His (N-terminal domain)	This paper	N/A
pET21-PfGRP94-His (N-terminal domain)	This paper	N/A
pET21-ScHSC82-His (N-terminal domain)	This paper	N/A
pSN054 conditional knockdown vector	Nasamu et al.^35^	N/A
p426 gRNA plasmid	DiCarlo et al.^47^	N/A
Software and algorithms		
GraphPad Prism 10	GraphPad Software	https://www.graphpad.com
Schrodinger Release 2024–3: Glide	Schrodinger, LLC	https://www.schrodinger.com
Schrodinger Release 2024–3: Prime/MM-GBSA	Schrodinger, LLC	https://www.schrodinger.com
Schrodinger Release 2024–3: LigPrep	Schrodinger, LLC	https://www.schrodinger.com
Schrodinger Release 2024–3: Protein Preparation Wizard	Schrodinger, LLC	https://www.schrodinger.com
PyMOL	Schrodinger, LLC	https://www.pymol.org
AlphaFold3	Google DeepMind	https://alphafoldserver.com
DESeq2	Love et al.45	https://bioconductor.org/packages/DESeq2
PoseEdit	Diedrich et al.28	https://poseedit.zbh.uni-hamburg.de

## Highlights

- Two HSP90 inhibitors targeting the same site show different resistance profiles
- Geldanamycin readily generates resistance; AUY-922 shows an exceptionally high barrier
- A resistance mutation paradoxically enhances rather than reduces drug binding
- Heat shock protein 90 knockdown differentially sensitizes parasites to the two inhibitors

## References

[1] Venkatesan P (2025). WHO world malaria report 2024. Lancet Microbe 6, 101073. 10.1016/j.lanmic.2025.101073. 39923782

[2] Haldar K, Bhattacharjee S, and Safeukui I (2018). Drug resistance in Plasmodium. Nat. Rev. Microbiol. 16, 156–170. 10.1038/nrmicro.2017.161. PMC6371404 29355852

[3] Rosenthal PJ, Asua V, and Conrad MD (2024). Emergence, transmission dynamics and mechanisms of artemisinin partial resistance in malaria parasites in Africa. Nat. Rev. Microbiol. 22, 373–384. 10.1038/s41579-024-01008-2. 38321292

[4] Siqueira-Neto JL, Wicht KJ, Chibale K, Burrows JN, Fidock DA, and Winzeler EA (2023). Antimalarial drug discovery: progress and approaches. Nat. Rev. Drug Discov. 22, 807–826. 10.1038/s41573-023-00772-9. PMC10543600 37652975

[5] Roy N, Nageshan RK, Ranade S, and Tatu U (2012). Heat shock protein 90 from neglected protozoan parasites. Biochim. Biophys. Acta. 1823, 707–711. 10.1016/j.bbamcr.2011.12.003. 22198098

[6] Ramdhave AS, Patel D, Ramya I, Nandave M, and Kharkar PS (2013). Targeting heat shock protein 90 for malaria. Mini. Rev. Med. Chem. 13, 1903–1920. 10.2174/13895575113136660094. 24070209

[7] Murillo-Solano C, Dong C, Sanchez CG, and Pizarro JC (2017). Identification and characterization of the antiplasmodial activity of Hsp90 inhibitors. Malar. J. 16, 292. 10.1186/s12936-017-1940-7. PMC5518105 28724415

[8] Posfai D, Eubanks AL, Keim AI, Lu K-Y, Wang GZ, Hughes PF, Kato N, Haystead TA, and Derbyshire ER (2018). Identification of Hsp90 Inhibitors with Anti-Plasmodium Activity. Antimicrob. Agents Chemother. 62, e0179917. 10.1128/AAC.01799-17. PMC5913967 29339390

[9] Janes J, Young ME, Chen E, Rogers NH, Burgstaller-Muehlbacher S, Hughes LD, Love MS, Hull MV, Kuhen KL, Woods AK, (2018). The ReFRAME library as a comprehensive drug repurposing library and its application to the treatment of cryptosporidiosis. Proc. Natl. Acad. Sci. USA 115, 10750–10755. 10.1073/pnas.1810137115. PMC6196526 30282735

[10] Southworth DR, and Agard DA (2008). Species-Dependent Ensembles of Conserved Conformational States Define the Hsp90 Chaperone ATPase Cycle. Mol. Cell 32, 631–640. 10.1016/j.molcel.2008.10.024. PMC2633443 19061638

[11] Li Z-N, and Luo Y (2023). HSP90 inhibitors and cancer: Prospects for use in targeted therapies (Review). Oncol. Rep. 49, 6. 10.3892/or.2022.8443. PMC9685368 36367182

[12] Felip E, Barlesi F, Besse B, Chu Q, Gandhi L, Kim S-W, Carcereny E, Sequist LV, Brunsvig P, Chouaid C, (2018). Phase 2 Study of the HSP-90 Inhibitor AUY922 in Previously Treated and Molecularly Defined Patients with Advanced Non–Small Cell Lung Cancer. J. Thorac. Oncol. 13, 576–584. 10.1016/j.jtho.2017.11.131. 29247830

[13] Swann J, Corey V, Scherer CA, Kato N, Comer E, Maetani M, Antonova-Koch Y, Reimer C, Gagaring K, Ibanez M, (2016). High-Throughput Luciferase-Based Assay for the Discovery of Therapeutics That Prevent Malaria. ACS Infect. Dis. 2, 281–293. 10.1021/acsinfecdis.5b00143. PMC4890880 27275010

[14] Fu Y, Tilley L, Kenny S, and Klonis N (2010). Dual labeling with a far red probe permits analysis of growth and oxidative stress in *P. falciparum* -infected erythrocytes. Cytometry. A. 77, 253–263. 10.1002/cyto.a.20856. 20091670

[15] Ottilie S, Luth MR, Hellemann E, Goldgof GM, Vigil E, Kumar P, Cheung AL, Song M, Godinez-Macias KP, Carolino K, (2022). Adaptive laboratory evolution in S. cerevisiae highlights role of transcription factors in fungal xenobiotic resistance. Commun. Biol. 5, 128. 10.1038/s42003-022-03076-7. PMC8837787 35149760

[16] Luth MR, Godinez-Macias KP, Chen D, Okombo J, Thathy V, Cheng X, Daggupati S, Davies H, Dhingra SK, Economy JM, (2024). Systematic in vitro evolution in *Plasmodium falciparum* reveals key determinants of drug resistance. Science 386, eadk9893. 10.1126/science.adk9893. PMC11809290 39607932

[17] Carrasquilla M, Drammeh NF, Rawat M, Sanderson T, Zenonos Z, Rayner JC, and Lee MCS (2022). Barcoding Genetically Distinct Plasmodium falciparum Strains for Comparative Assessment of Fitness and Antimalarial Drug Resistance. mBio 13, e0093722. 10.1128/mbio.00937-22. PMC9600763 35972144

[18] Lim MY-X, LaMonte G, Lee MCS, Reimer C, Tan BH, Corey V, Tjahjadi BF, Chua A, Nachon M, Wintjens R, (2016). UDP-galactose and acetyl-CoA transporters as Plasmodium multidrug resistance genes. Nat. Microbiol. 1, 16166. 10.1038/nmicrobiol.2016.166. PMC5575994 27642791

[19] Millson SH, Chua C-S, Roe SM, Polier S, Solovieva S, Pearl LH, Sim T-S, Prodromou C, and Piper PW (2011). Features of the Streptomyces hygroscopicus HtpG reveal how partial geldanamycin resistance can arise with mutation to the ATP binding pocket of a eukaryotic Hsp90. FASEB J. 25, 3828–3837. 10.1096/fj.11-188821. 21778327

[20] Bridgford JL, Xie SC, Cobbold SA, Pasaje CFA, Herrmann S, Yang T, Gillett DL, Dick LR, Ralph SA, Dogovski C, (2018). Artemisinin kills malaria parasites by damaging proteins and inhibiting the proteasome. Nat. Commun. 9, 3801. 10.1038/s41467-018-06221-1. PMC6143634 30228310

[21] LaMonte GM, Rocamora F, Marapana DS, Gnädig NF, Ottilie S, Luth MR, Worgall TS, Goldgof GM, Mohunlal R, Santha Kumar TR, (2020). Pan-active imidazolopiperazine antimalarials target the Plasmodium falciparum intracellular secretory pathway. Nat. Commun. 11, 1780. 10.1038/s41467-020-15440-4. PMC7156427 32286267

[22] Duffey M, Blasco B, Burrows JN, Wells TNC, Fidock DA, and Leroy D (2021). Assessing risks of Plasmodium falciparum resistance to select next-generation antimalarials. Trends Parasitol. 37, 709–721. 10.1016/j.pt.2021.04.006. PMC8282644 34001441

[23] Phillips MA, Lotharius J, Marsh K, White J, Dayan A, White KL, Njoroge JW, El Mazouni F, Lao Y, Kokkonda S, (2015). A long-duration dihydroorotate dehydrogenase inhibitor (DSM265) for prevention and treatment of malaria. Sci. Transl. Med. 7, 296ra111. 10.1126/scitranslmed.aaa6645. PMC4539048 26180101

[24] Whitesell L, Robbins N, Huang DS, McLellan CA, Shekhar-Guturja T, LeBlanc EV, Nation CS, Hui R, Hutchinson A, Collins C, (2019). Structural basis for species-selective targeting of Hsp90 in a pathogenic fungus. Nat. Commun. 10, 402. 10.1038/s41467-018-08248-w. PMC6345968 30679438

[25] Prodromou C, Roe SM, O’Brien R, Ladbury JE, Piper PW, and Pearl LH (1997). Identification and structural characterization of the ATP/ADP-binding site in the Hsp90 molecular chaperone. Cell 90, 65–75. 10.1016/s0092-8674(00)80314-1. 9230303

[26] Suzuki Y, St Onge RP, Mani R, King OD, Heilbut A, Labunskyy VM, Chen W, Pham L, Zhang LV, Tong AHY, (2011). Knocking out multigene redundancies via cycles of sexual assortment and fluorescence selection. Nat. Methods 8, 159–164. 10.1038/nmeth.1550. PMC3076670 21217751

[27] Hekkelman ML, De Vries I, Joosten RP, and Perrakis A (2023). AlphaFill: enriching AlphaFold models with ligands and cofactors. Nat. Methods 20, 205–213. 10.1038/s41592-022-01685-y. PMC9911346 36424442

[28] Corbett KD, and Berger JM (2010). Structure of the ATP-binding domain of *Plasmodium falciparum* Hsp90. Proteins 78, 2738–2744. 10.1002/prot.22799. PMC2927708 20635416

[29] Diedrich K, Krause B, Berg O, and Rarey M (2023). PoseEdit: enhanced ligand binding mode communication by interactive 2D diagrams. J. Comput. Aided. Mol. Des. 37, 491–503. 10.1007/s10822-023-00522-4. PMC10440272 37515714

[30] Özen A, and Schiffer CA (2017). Substrate-Envelope-Guided Design of Drugs with a High Barrier to the Evolution of Resistance. In Handbook of Antimicrobial Resistance, Berghuis A, Matlashewski G, Wainberg MA, and Sheppard D, eds. (Springer New York), pp. 149–173. 10.1007/978-1-4939-0694-9_9.

[31] Kim J, Felts S, Llauger L, He H, Huezo H, Rosen N, and Chiosis G (2004). Development of a Fluorescence Polarization Assay for the Molecular Chaperone Hsp90. SLAS Discovery 9, 375–381. 10.1177/1087057104265995. 15296636

[32] Friesner RA, Banks JL, Murphy RB, Halgren TA, Klicic JJ, Mainz DT, Repasky MP, Knoll EH, Shelley M, Perry JK, (2004). Glide: A New Approach for Rapid, Accurate Docking and Scoring. 1. Method and Assessment of Docking Accuracy. J. Med. Chem. 47, 1739–1749. 10.1021/jm0306430. 15027865

[33] Genheden S, and Ryde U (2015). The MM/PBSA and MM/GBSA methods to estimate ligand-binding affinities. Expert Opin. Drug Discov. 10, 449–461. 10.1517/17460441.2015.1032936. PMC4487606 25835573

[34] Ganesan SM, Falla A, Goldfless SJ, Nasamu AS, and Niles JC (2016). Synthetic RNA–protein modules integrated with native translation mechanisms to control gene expression in malaria parasites. Nat. Commun. 7, 10727. 10.1038/ncomms10727. PMC4773503 26925876

[35] Nasamu AS, Falla A, Pasaje CFA, Wall BA, Wagner JC, Ganesan SM, Goldfless SJ, and Niles JC (2021). An integrated platform for genome engineering and gene expression perturbation in Plasmodium falciparum. Sci. Rep. 11, 342. 10.1038/s41598-020-77644-4. PMC7801740 33431920

[36] Li Y, Zou L, Li Q, Haibe-Kains B, Tian R, Li Y, Desmedt C, Sotiriou C, Szallasi Z, Iglehart JD, (2010). Amplification of LAPTM4B and YWHAZ contributes to chemotherapy resistance and recurrence of breast cancer. Nat. Med. 16, 214–218. 10.1038/nm.2090. PMC2826790 20098429

[37] Muzenda FL, Stofberg ML, Mthembu W, Achilonu I, Strauss E, and Zininga T (2025). Characterization and Inhibition of the Chaperone Function of *Plasmodium falciparum* Glucose-Regulated Protein 94 kDa ( Pf Grp94 ). Proteins 93, 957–971. 10.1002/prot.26779. PMC11968560 39670568

[38] Stofberg ML, Muzenda FL, Achilonu I, Strauss E, and Zininga T (2025). *In silico* screening of selective ATP mimicking inhibitors targeting the *Plasmodium falciparum* Grp94. J. Biomol. Struct. Dyn. 43, 6214–6225. 10.1080/07391102.2024.2329304. 38498364

[39] Yang T, Ottilie S, Istvan ES, Godinez-Macias KP, Lukens AK, Baragaña B, Campo B, Walpole C, Niles JC, Chibale K, (2021). MalDA, Accelerating Malaria Drug Discovery. Trends Parasitol. 37, 493–507. 10.1016/j.pt.2021.01.009. PMC8261838 33648890

[40] De Lera Ruiz M, Favuzza P, Guo Z, Zhao L, Hu B, Lei Z, Zhan D, Murgolo N, Boyce CW, Vavrek M, (2022). The Invention of WM382, a Highly Potent PMIX/X Dual Inhibitor toward the Treatment of Malaria. ACS Med. Chem. Lett. 13, 1745–1754. 10.1021/acsmedchemlett.2c00355. PMC9661708 36385924

[41] Deni I, Stokes BH, Ward KE, Fairhurst KJ, Pasaje CFA, Yeo T, Akbar S, Park H, Muir R, Bick DS, (2023). Mitigating the risk of antimalarial resistance via covalent dual-subunit inhibition of the Plasmodium proteasome. Cell Chem. Biol. 30, 470–485.e6. 10.1016/j.chembiol.2023.03.002. PMC10198959 36963402

[42] Makhoba XH, Viegas C, Mosa RA, Viegas FPD, and Pooe OJ (2020). Potential Impact of the Multi-Target Drug Approach in the Treatment of Some Complex Diseases. Drug Des. Devel. Ther. 14, 3235–3249. 10.2147/DDDT.S257494. PMC7440888 32884235

[43] Yang Y, Tang T, Li X, Michel T, Ling L, Huang Z, Mulaka M, Wu Y, Gao H, Wang L, (2021). Design, synthesis, and biological evaluation of multiple targeting antimalarials. Acta Pharm. Sin. B 11, 2900–2913. 10.1016/j.apsb.2021.05.008. PMC8463279 34589403

[44] Lawong A, Gahalawat S, Ray S, Ho N, Han Y, Ward KE, Deng X, Chen Z, Kumar A, Xing C, (2024). Identification of potent and reversible piperidine carboxamides that are species-selective orally active proteasome inhibitors to treat malaria. Cell Chem. Biol. 31, 1503–1517.e19. 10.1016/j.chembiol.2024.07.001. PMC11531662 39084225

[45] Mansfield CR, Quan B, Chirgwin ME, Eduful B, Hughes PF, Neveu G, Sylvester K, Ryan DH, Kafsack BFC, Haystead TAJ, (2024). Selective targeting of Plasmodium falciparum Hsp90 disrupts the 26S proteasome. Cell Chem. Biol. 31, 729–742.e13. 10.1016/j.chembiol.2024.02.008. PMC11031320 38492573

[46] Tye MA, Payne NC, Johansson C, Singh K, Santos SA, Fagbami L, Pant A, Sylvester K, Luth MR, Marques S, (2022). Elucidating the path to Plasmodium prolyl-tRNA synthetase inhibitors that overcome halofuginone resistance. Nat. Commun. 13, 4976. 10.1038/s41467-022-32630-4. PMC9403976 36008486

[47] DiCarlo JE, Norville JE, Mali P, Rios X, Aach J, and Church GM (2013). Genome engineering in Saccharomyces cerevisiae using CRISPR-Cas systems. Nucleic Acids Res. 41, 4336–4343. 10.1093/nar/gkt135. PMC3627607 23460208

[48] Adjalley S, and Lee MCS (2022). CRISPR/Cas9 Editing of the Plasmodium falciparum Genome. In Malaria Immunology Methods in Molecular Biology, Jensen ATR and Hviid L, eds. (Springer US), pp. 221–239. 10.1007/978-1-0716-2189-9_17. 35881349

[49] Love MI, Huber W, and Anders S (2014). Moderated estimation of fold change and dispersion for RNA-seq data with DESeq2. Genome Biol. 15, 550. 10.1186/s13059-014-0550-8. PMC4302049 25516281

[50] (2024). Schrödinger Release 2024–3: Prime (Schrödinger, LLC).

[51] (2024). Schrödinger Release 2024–3: Protein Prep (Schrödinger, LLC).

[52] (2024). Schrödinger Release 2024–3 (LigPrep, Schrödinger, LLC).

[53] (2024). Schrödinger Release 2024–3: Glide (Schrödinger, LLC).

[54] (2024). Schrödinger Release 2024–3: Prime/MM-GBSA (Schrödinger, LLC).

[55] Deitsch K, Driskill C, and Wellems T (2001). Transformation of malaria parasites by the spontaneous uptake and expression of DNA from human erythrocytes. Nucleic Acids Res. 29, 850–853. 10.1093/nar/29.3.850. PMC30384 11160909

