# Supplementary material for: Plasmodium falciparum HSP90 inhibitors show divergent resistance despite a shared ATP-binding site
Source: Cell Rep. Author manuscript; Available in PMC 2026 Sep 19. (PMC13589290; doi:10.1016/j.celrep.2026.117664)
Supplement: Supplementary file 1 [file NIHMS2199965-supplement-1.pdf]

Cell Reports, Volume *45*

Supplemental information

Plasmodium falciparum HSP90 inhibitors show divergent resistance despite a shared ATP-binding site

Fu-Hsuan Ko, Amanda K. Lukens, Mariana Laureano de Souza, Alex Foy, Jason Hsiao, Tolla Ndiaye, John Okombo, Tomas Yeo, Heekuk Park, Anne-Catrin Uhlemann, Nonlawat Boonyalai, Krittikorn Kumpornsin, Gareth Girling, Charisse Pasaje, Luiz Godoy, Priyan Kapoor, Gregory L. Durst, Sabine Ottilie, Arnab Chatterjee, Jacquin Niles, Marcus Lee, David A. Fidock, Dyann F. Wirth, and Elizabeth A. Winzeler

Supplemental information

Figure S1

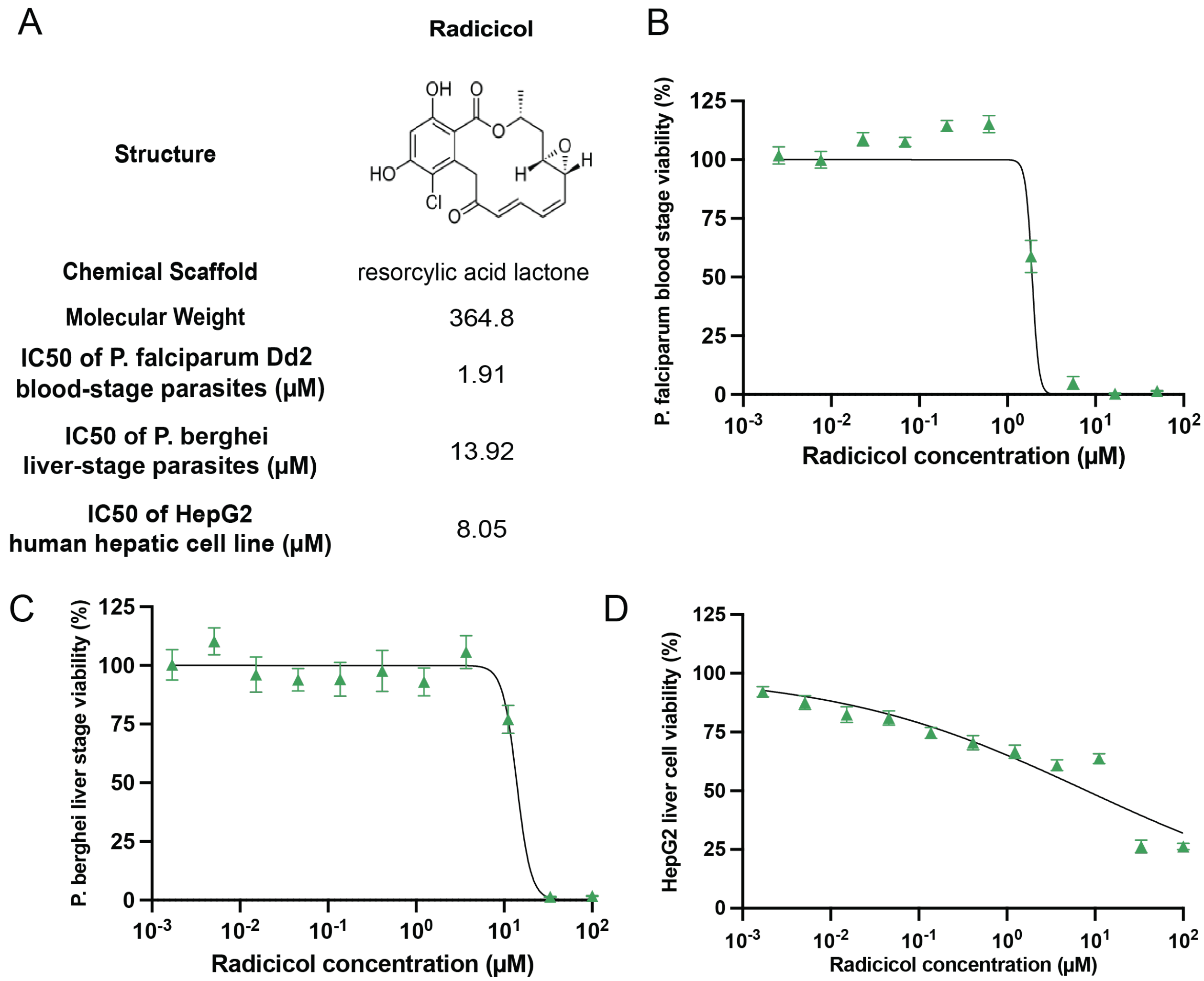

Figure S1. Antimalarial characterization of radicicol as an HSP90 inhibitor

(A) Chemical structure and biological activity profile of radicicol showing its resorcylic acid lactone scaffold and IC_{50} values against different parasite stages and human cells. **(B)** Dose-response curve of radicicol against *P. falciparum* Dd2 blood-stage parasites. **(C)** Dose-response curve of radicicol against *P. berghei* liver-stage parasites. **(D)** Cytotoxicity assessment of radicicol in HepG2 human hepatocytes. Data are represented as mean ± SEM of biological replicates.

Figure S2

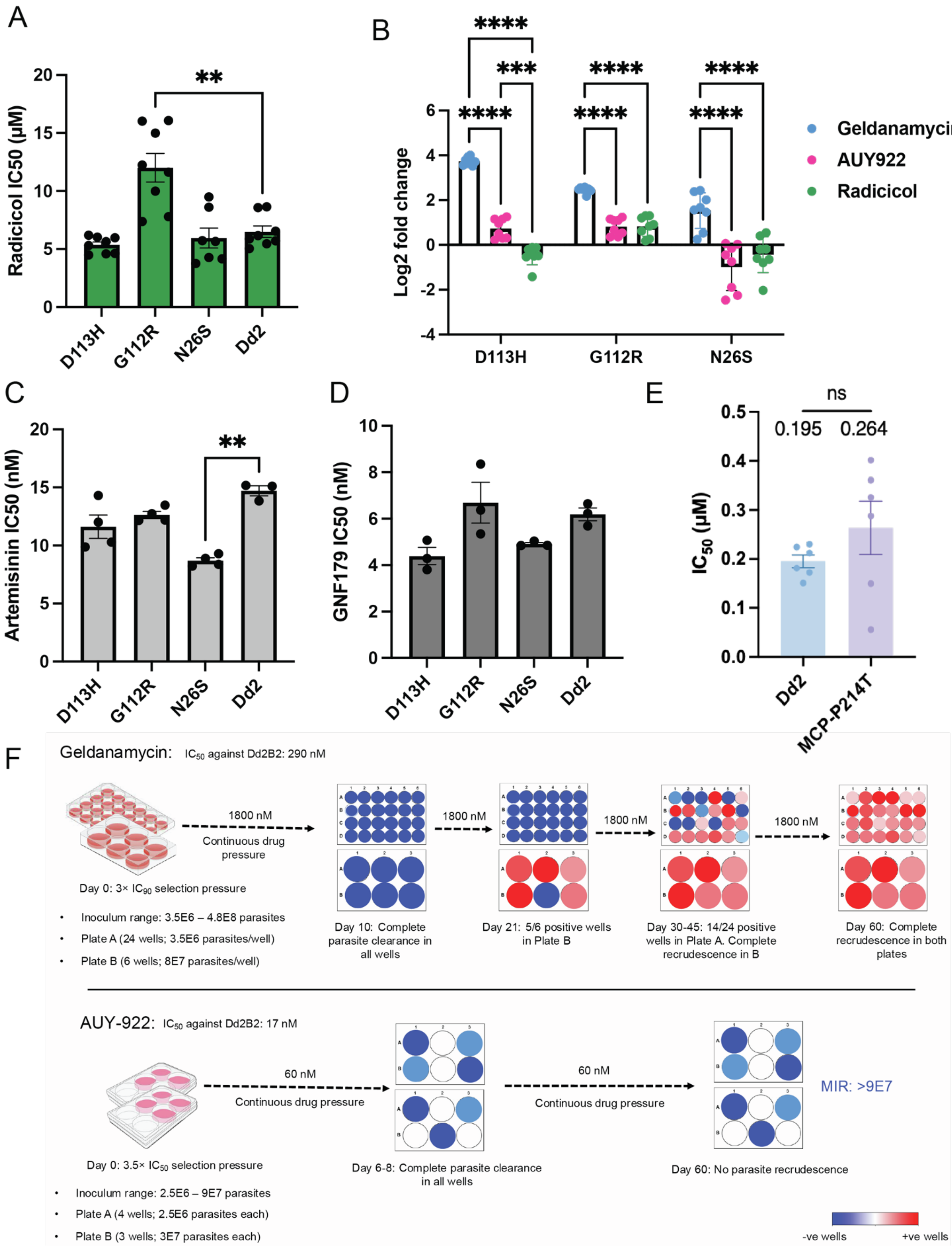

Figure S2. Cross-resistance characterization of geldanamycin-resistant clones and Minimum Inoculum for Resistance analysis

(A) IC_{50} values of geldanamycin-resistant clones against radicicol. (B) Log_2 fold-change analysis of geldanamycin-resistant mutations (D113H, G112R, N26S) against geldanamycin (blue), AUY-922 (magenta), and radicicol (green). **(C)**IC_{50} values of geldanamycin-resistant clones against artemisinin. **(D)** IC_{50} values of geldanamycin-resistant clones against GNF179. **(E)** IC50 values of the Dd2 parasite carrying MCP (mitochondrial carrier protein, PF3D7_0908800) P214T mutation against geldanamycin, showing no significant difference in drug sensitivity compared to the parental Dd2 line. **(F)** Minimum Inoculum for Resistance (MIR) assay results. Top: Geldanamycin MIR assay progression from Day 0 to Day 60. Bottom: AUY-922 MIR assay showing no recrudescence (MIR $>9\times10^7$). Blue circles represent negative wells, red circles represent positive wells, white circles represent wells not tested. Data are represented as mean ± SEM. *$p < 0.05$, ***$p < 0.001$, ****$p < 0.0001$ by Brown-Forsythe and Welch ANOVA with Dunnett's T3 multiple comparisons test (A, B, C, and D) or by unpaired Student's t test with Welch's correction (E).

Figure S3

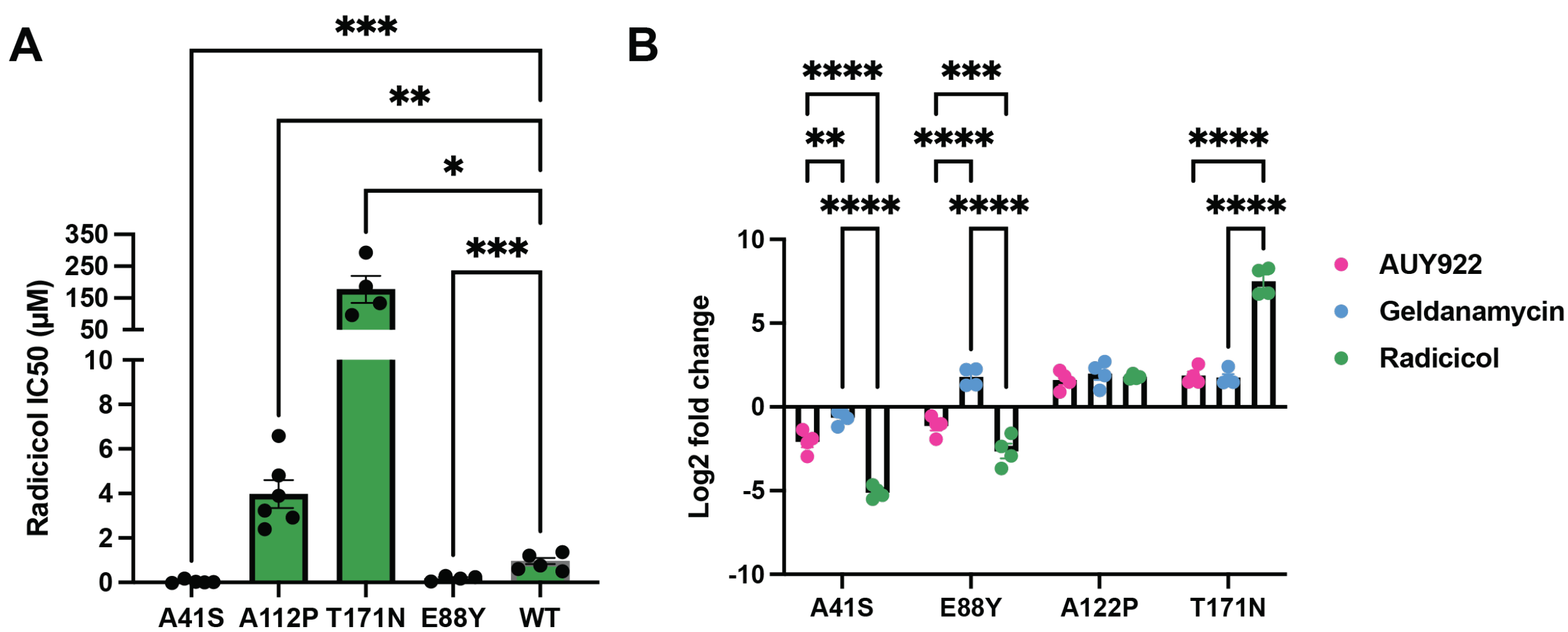

Figure S3. Cross-species comparison of HSP90 inhibitor sensitivity in *S. cerevisiae*

(A) IC_{50} values of CRISPR-engineered *S. cerevisiae* HSP90 mutations against radicicol. **(B)** IC_{50} fold-changes of CRISPR-engineered *S. cerevisiae* HSP90 mutations against AUY-922, geldanamycin, and radicicol, demonstrating mutation-specific resistance and sensitivity patterns across the three HSP90 inhibitors. Data are represented as mean ± SEM. *$p < 0.05$, ***$p < 0.001$, ****$p < 0.0001$ by Brown-Forsythe and Welch ANOVA with Dunnett's T3 multiple comparisons test.

Figure S4

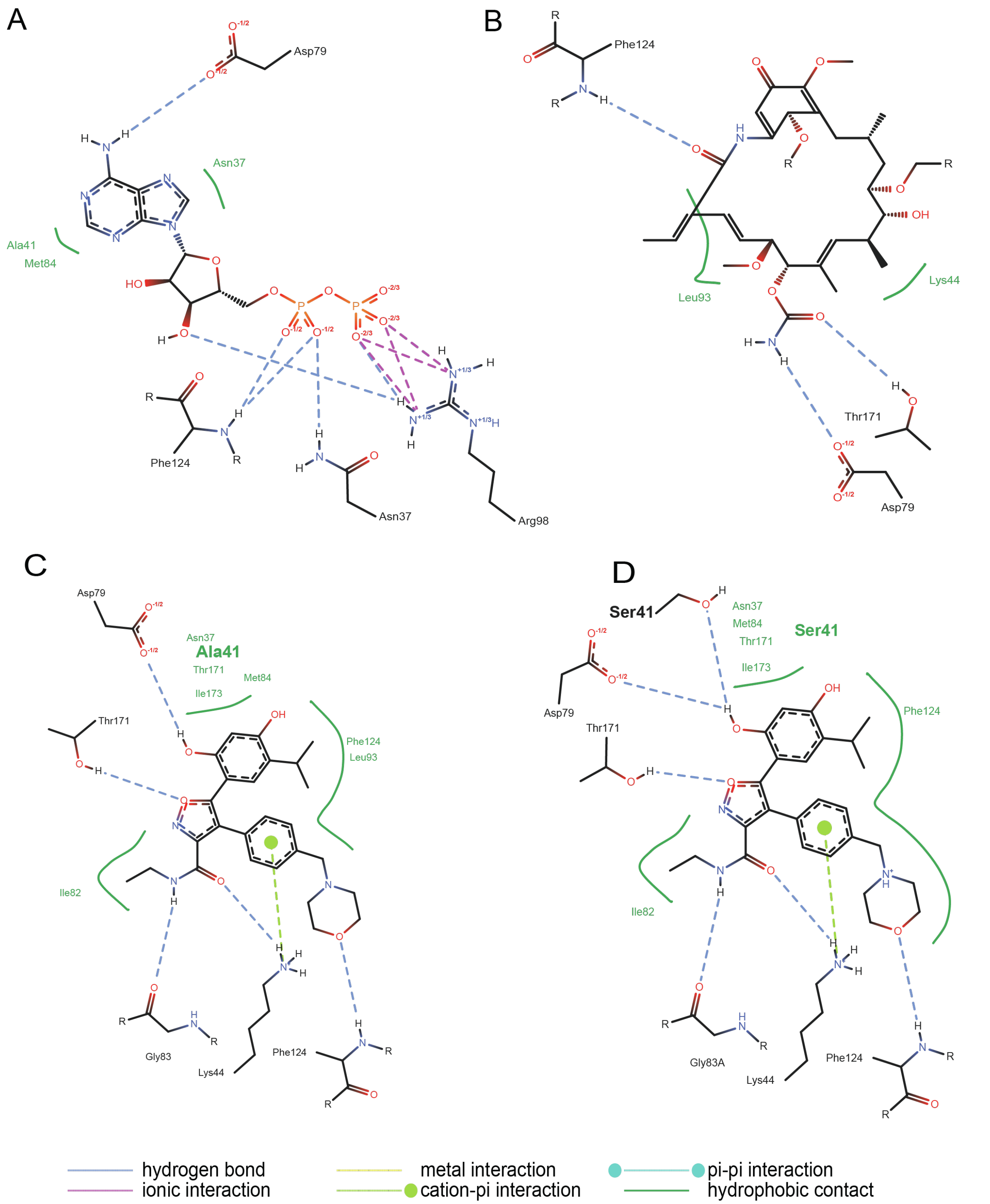

Figure S4. PoseEdit ligand interaction analysis

(A) PoseEdit interaction diagram of ADP binding to PfHSP90. **(B)** PoseEdit interaction diagram of geldanamycin binding to PfHSP90. **(C)** PoseEdit interaction diagram of AUY-922 binding to wild-type PfHSP90 with Ala41 highlighted. **(D)**PoseEdit interaction diagram of AUY-922 binding to PfHSP90 A41S mutant with Ser41 highlighted.

Figure S5

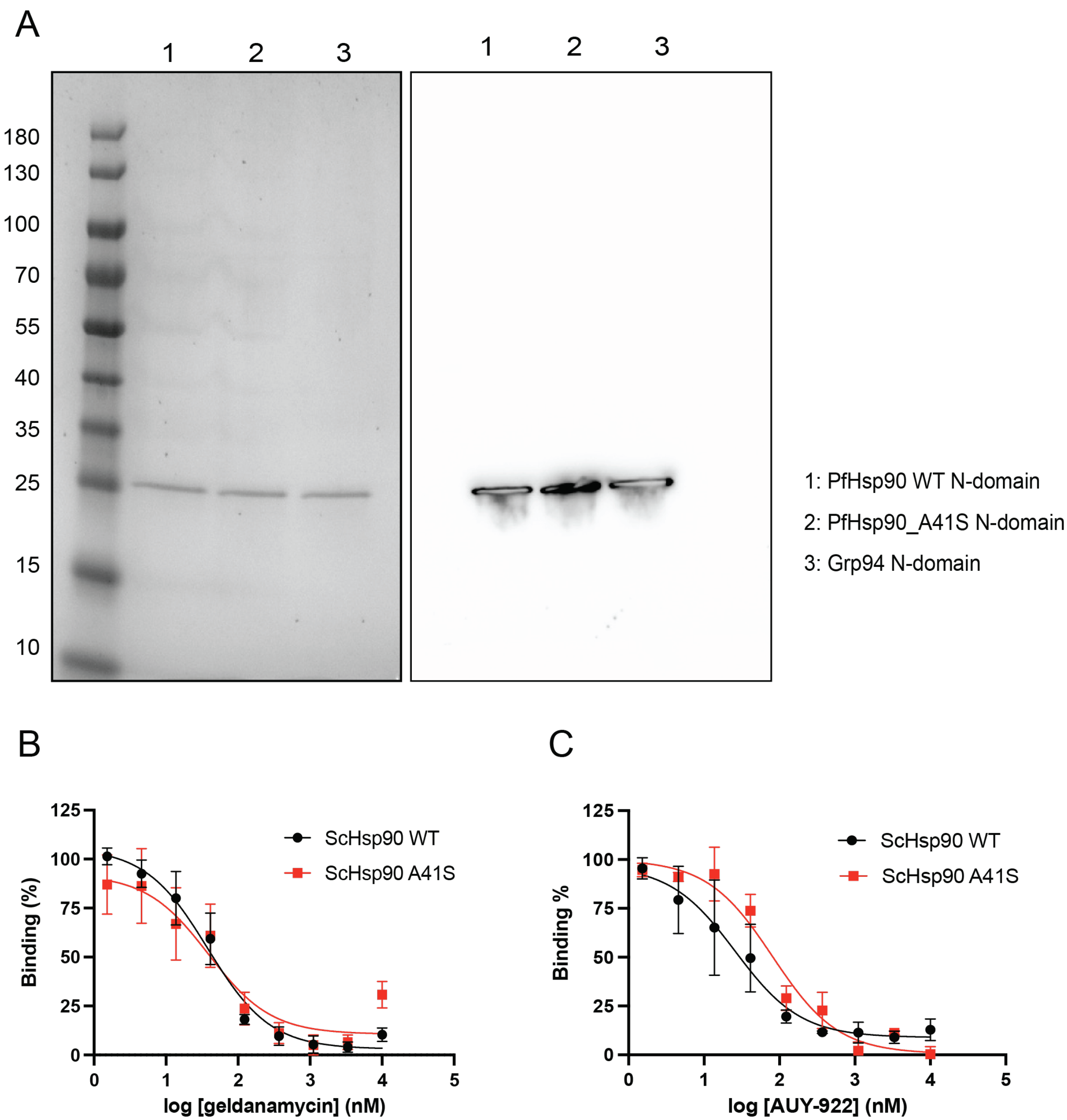

Figure S5. Purification of recombinant *P. falciparum* HSP90 proteins and binding characterization of yeast HSC82 A41S mutation

(A) SDS-PAGE analysis showing purified proteins. Left: Coomassie blue staining. Right: Anti-His Western blot confirming successful purification of PfHsp90 wild-type N-domain, PfHSP90 A41S N-domain, and PfGRP94 N-domain. **(B)** Fluorescence polarization competition assays of wild-type and A41S yeast HSC82 N-terminal domain against geldanamycin, showing no difference in binding affinity between the two variants. **(C)** Fluorescence polarization competition assays of wild-type and A41S yeast HSC82 N-terminal domain against AUY-922, showing reduced binding affinity in the A41S variant compared to wild type. Data are represented as mean ± SEM of biological replicates.

Figure S6

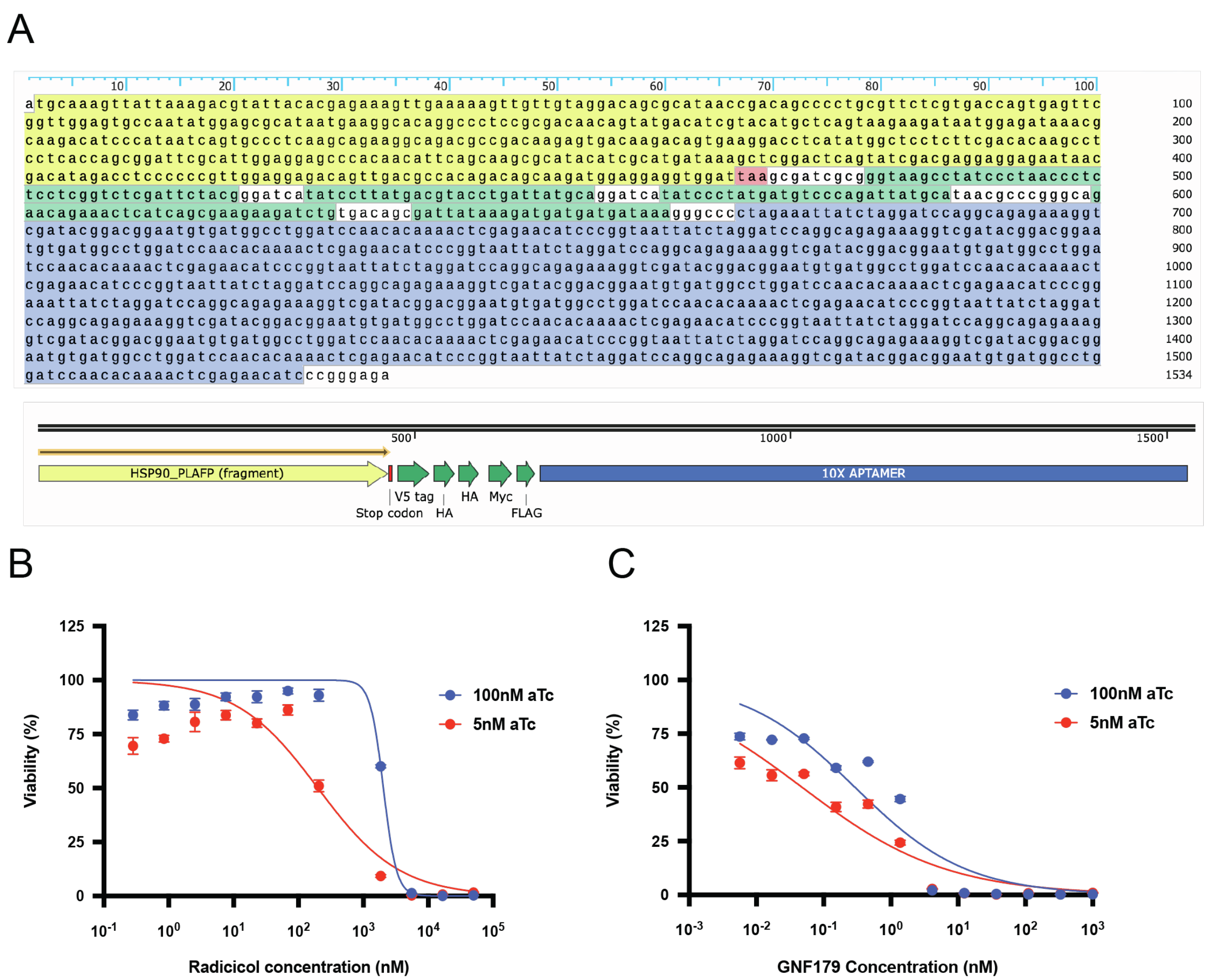

Figure S6. Confirmation of PfHSP90 conditional knockdown cassette integration and drug sensitivity profiling under knockdown conditions

(A) Sequencing confirmation of successful aptamer cassette integration downstream of the PfHSP90 3' end. The PfHSP90 coding sequence is highlighted in yellow, the introduced stop codon in red, and the 10× aptamer array in blue. Orange arrows indicate the translated open reading frame. **(B)** IC_{50} shift assay for radicicol under HSP90 knockdown conditions comparing 5 nM vs 100 nM aTc treatment. **(C)** IC_{50} shift assay for GNF179 under HSP90 knockdown conditions comparing 5 nM vs 100 nM aTc treatment. Data are represented as mean ± SEM of biological replicates.
